# Water Is Cool: Advanced Phonon Dynamics in Ice Ih and Ice XI via Machine Learning Potentials and Quantum Nuclear Vibrations

**DOI:** 10.1021/acs.jctc.4c01582

**Published:** 2025-02-07

**Authors:** Aleksandar Živković, Umberto Terranova, Nora H. de Leeuw

**Affiliations:** † Department of Earth Sciences, Utrecht University, Princetonlaan 8a, 3584CB Utrecht, The Netherlands; ‡ Faculty of Medicine and Health Sciences, Crewe Campus, University of Buckingham, Crewe CW1 5DU, United Kingdom; § School of Chemistry, University of Leeds, Leeds LS2 9JT, United Kingdom; ∥ Institute for Inorganic Chemistry, Christian-Albrechts-Universität zu Kiel, Max-Eyth-Str. 2, 24118 Kiel,Germany

## Abstract

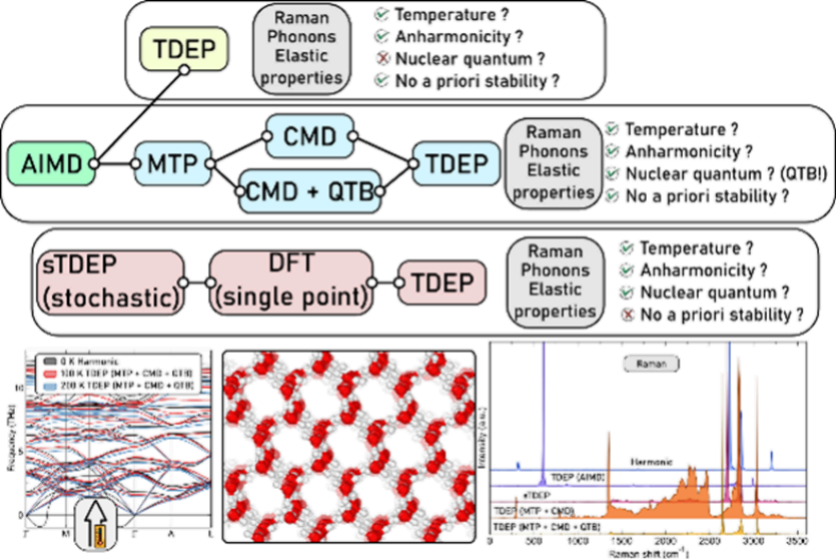

Low-dimensional water, despite the relative simplicity of its constituents, exhibits a vast range of phenomena that are of central importance in natural sciences. A large number of bulk as well as nanoscale polymorphs offer engineering possibilities for technological applications such as desalinization, drug delivery, or biological interfacing. However, little is known about the stability of such structures. Therefore, in this study, we employ an array of state-of-the-art computational techniques to study the vibrational properties of ice Ih and XI in their bulk and thin film forms in order to elucidate their structural stability and dynamic behavior. An efficient workflow, consisting of quantum mechanical simulations (based on density functional theory) and machine learning interatomic potentials (MTPs) coupled to temperature-dependent effective potentials (TDEP) and classical molecular dynamics, was verified necessary to capture the temperature-dependent stabilization of the phonons in bulk ice Ih and XI. Anharmonicity and nuclear quantum effects, incorporated in an efficient way through a quantum thermal bath technique, were found crucial to dynamically stabilize low-frequency lattice modes and high-frequency vibrational stretching involving hydrogen. We have identified three novel thin film structures that retain their stability up to at least 250 K and have shed light on their phonon characteristics. In addition, our examination of the Raman spectrum of ice underscores the shortcomings of predicting vibrational properties when relying entirely on the harmonic approximation or purely anharmonic effects. The corrected redistribution of vibrational intensities is found to be achieved only upon inclusion of quantum nuclear vibrations. This was found to be even more crucial for low-dimensional thin film (2D) structures. Overall, our findings demonstrate the significance of joining advanced computational methodologies in unraveling the intricate vibrational dynamics of crystalline ice materials, offering valuable insights into their thermodynamic and structural properties. Furthermore, we suggest a procedure based on MTPs coupled to a quantum thermal bath for the computationally efficient probing of nuclear effects in ice structures, although equally applicable to any other system.

## Introduction

Water and ice, ubiquitous compounds on Earth, exhibit a rich array of behaviors that are crucial for the understanding of a variety of natural and technological processes. From the fundamental chemistry of aqueous solutions to the dynamics of planetary ice caps, the study of water and ice spans a wide range of disciplines and applications. Understanding the properties of water and ice is not only essential for elucidating phenomena on our planet but also has implications for space research, where water ice plays a fundamental role in planetary science and astrobiology.^1^ Moreover, water’s unique behavior in confined environments, such as those found in nanomaterials or at interfaces, holds promise for applications in fields ranging from nanofluidics to biotechnology.^2^

Studying water and ice interfaces is crucial for our understanding of a wide range of phenomena from catalytic processes to biomolecular interactions. Water’s interactions at interfaces, spanning the hydrophobic to hydrophilic spectrum, can significantly influence the behavior of molecules and materials in contact with an aqueous environment.^3^ Vibrational dynamics of biomolecules in an aqueous environment were found to have a strong correlation with water molecules by forming bridging networks of H-bonds.^4^ The literature pertaining to the properties and behaviors of ice and water compounds is extensive and encompasses a diverse array of studies spanning numerous disciplines. We outline a small subset in the following paragraphs.

Ice Ih consists of oxygen atoms in a regular hexagonal lattice with hydrogen atoms distributed at “*random*”, but subject to the Bernal–Fowler ice rules, often called proton disorder in literature.^5^ The structural diversity of ice stems from the tetrahedrally coordinated oxygen atoms and the multitude or arrangements they form.^6^ Below a temperature of around 70 K, ice becomes proton ordered and ferroelectric while retaining the hexagonal symmetry (albeit with a change in symmetry from 6_3_/mmc to Cmc2_1_)—a phase known as ice XI.^7^ Pan et al. studied the basal and prism surfaces of ice Ih and explored the degree of proton disorder when compared to the bulk using density functional theory (DFT) simulations.^8^ They noticed a strong dependence of the surface energy on the surface proton order and discussed additional parameters (describing the surface OH dangling group arrangement), which should be added to simulations of ice Ih surfaces in addition to the Bernal–Fowler–Pauling rules. Ikutaro Hamada used a van der Waals density functional to study ice Ih and demonstrated a predicted binding energy comparable to high level quantum chemistry calculations.^9^ Zhao et al.^10^ presented a comprehensive overview of molecular dynamics simulations of structures and phase behavior of highly confined water, ice, amorphous ice, and clathrate in slit graphene nanopores. The stability of four high-density monolayer ice structures in a vacuum was demonstrated, although their frequency calculations were not reported explicitly.

Morawietz and co-workers used ab initio (RPBE and BLYP) calculations paired with neural network potentials to demonstrate that the vdW interactions are crucial for the formation of water’s density maximum and negative melting volume.^11^ For a comprehensive overview on the topic of surface premelting, where frozen water has a quasi-liquid layer at its surface, see the review by Slater and Michaelides.^12^ Furthermore, Fumagalli et al. evaluated the dielectric constant of interfacial water confined between atomically flat walls separated by various distances up to 1 nm.^13^ They revealed that the dielectric constant of confined water strongly changes with a decreasing channel height. The out-of-plane dielectric constant (ε_⊥_) converged to a limiting value of  for interlayer spacing smaller than 2 nm, which equals to a resolution of only a few layers of water inside the channels. This is assigned to a suppressed dipole rotational contribution to the dielectric constant, at least in the direction perpendicular to the atomic planes of the confining channels.

Artemov studied complete dielectric spectra of ice and water by using an oscillator model and was able to provide a good spectral description of both compounds up to 10 THz.^14^ As an alternative to the rotating H_2_O dipoles, the authors suggested that the polarization arises due to local fluctuations of ions by spontaneous exchange of excess protons with neutral molecules.

The literature reveals an ever-increasing complexity and abundance of effects that water and ice show in their bulk or low-dimensional form. However, for any computational model to obtain sensible results, it is important to capture coexisting effects simultaneously, which range from dispersion interactions to proton disorder, all while being able to describe the relative stability of competing phases of the prolific phase diagram of ice.

For ice, the phase of water containing a periodically repeating crystal lattice, it is of paramount importance to understand the underlying lattice dynamics, as it provides invaluable information about the stability and behavior of ice polymorphs. Phonons, the quantized vibrations that govern the lattice dynamics of crystalline solids, play a pivotal role in determining the thermal, mechanical, and transport properties of the materials. Even so, given the light proton mass of the ice constituents, anharmonic effects (all those not captured by the harmonic model) and nuclear quantum effects (NQEs) are known to be significant in aqueous systems. Understanding phonons in ice phases is therefore essential for the development of realistic computational models and simulations, ensuring that the reported results are physically meaningful and applicable to real-world scenarios. Thus far, this information is missing in the existing literature.

In this work, using a systematic and tractable workflow approach, we have simulated the 3D bulk structure of ice in the Ih and XI phases together with their 2D surfaces of varying thicknesses and terminations. To capture the necessary physics and chemistry of these phases, a synergistic range of theoretical methods is employed, starting from density functional theory coupled with machine learned interatomic potentials, the temperature dependent effective potential method, and classical molecular dynamics. In comparison to the existing literature, where often 2D structures of various origins and thicknesses can be found, we have used a systematic approach to obtain well-defined, reproducible, and tractable surface structures of ice Ih and XI with varying thicknesses. To those we refer as thin films as they do not exceed more than 1.5 nm in the nonperiodic direction.

In this work, we attempt to answer the following questions:

(i) Which array of methods is necessary and capable of accurately reproducing the phonon properties of ice Ih and XI?
Iii) Are thin film ice Ih and XI structures stable in their 2D form?
(iii) What role do anharmonicity and NQEs play in the Raman spectra of these ice structures?

## Computational Details

### First-Principles

Density functional theory calculations were performed by using the Vienna ab initio simulation package (VASP) with the projector augmented-wave (PAW) method.^15−17^ For the PAW potentials, the valence electronic configurations used were s^2^p^4^ for O and s^1^ for H. An energy cutoff of 600 eV was used to truncate the plane-wave expansion. The general gradient approximation (GGA) for the exchange-correlation (XC) functional was employed within the Perdew–Burke–Ernzerhof parametrization (PBE),^18^ where long-range dispersion corrections were included using the D2 approach of Grimme et al.^19^ The conjugate gradient method was used for structural optimizations with static ground-state DFT, with the total energy and force convergence criteria set to at least 10^–7^ eV and 0.002 eV/Å, respectively. The Brillouin zone was sampled using 5 × 5 × 5 and 9 × 9 × 7 Monkhorst–Pack meshes for the bulk ice Ih and ice XI phases, respectively.^20^

### Phonons

Lattice dynamics calculations in the harmonic approximation to construct and evaluate the dynamical matrix were carried out using the Phonopy package.^21−23^ The supercell finite displacement method was used to construct the force constants on top of forces obtained with DFT from VASP. Long-range dipole–dipole interactions were taken into account through the so-called nonanalytical term correction model (NAC).^24^ Phonon density of states (DOS) curves were obtained by interpolating the phonon frequencies onto a uniform 11 × 11 × 11 and 15 × 15 × 15 Γ-centered q-point mesh for ice Ih and XI, respectively, together with the linear tetrahedron method for the Brillouin-zone integration. The phonon dispersions were obtained by interpolating the phonon frequencies along lines of q-points passing between the high-symmetry points in the Brillouin zones of the primitive unit cells. For cross-validation, Phonopy calculations of ice XI in the 4 × 4 × 2 supercell at the Γ-point were performed using CP2K as a force generator.^25,26^ We adopted the PBE exchange-correlation functional, the Goedecker, Teter, and Hutter (GTH) Gaussian basis set,^27−30^ and an energy cutoff of 800 Ry.

Vibrational frequency calculations were also performed using the all-electron code CRYSTAL17^31,32^ with the global hybrid B3YLP exchange-correlation functional approximation.^33,34^ There, a basis set based on a linear combination of atomic orbitals (LCAO) is employed, where atoms are described using basis sets reported in earlier literature: Peintinger–Oliveira–Bredow double-ζ-valence + polarization (pob-DVZP-rev2) for O and triple-ζ-valence + polarization (pob-TVZP-rev2) for H.^35^ Relative infrared and Raman intensities were computed analytically, based on coupled-perturbed Hartree–Fock/Kohn–Sham (CPHF/KS) treatments implemented in the code.^36−38^

### Anharmonic Lattice Dynamics

To go beyond the harmonic approximation and probe anharmonicity effects in the chosen materials, we have calculated interatomic force-constants at different temperatures using the state-of-the-art temperature-dependent effective potential (TDEP) method.^39−42^ This approach determines interatomic force-constants by minimizing the difference between a model system and ab initio calculated forces. Here we use three different ways of sampling the potential energy surface to generate the required force constants:

(i) Via explicit ab initio molecular dynamics (MD) simulations (referred to as TDEP (AIMD)). Ab-initio molecular dynamics simulations (AIMD) were performed using VASP with a time-step of 0.5 fs. We used a 2 × 2 × 2 supercell of ice Ih and a 4 × 4 × 2 supercell of ice XI for the AIMD simulations in the NVT ensemble (Brillouin zone integrated only at the Γ-point). An initial thermalization of at least 2.5 ps was performed before a sampling period of at least 5 ps that was then used to extract the force constants with TDEP.
(ii) Via stochastic sampling (referred to as stochastic TDEP–sTDEP^43^) with a series of first-principles simulations which are repeated until self-consistency is reached. Here the sampling was done with 50 snapshots (supercells) at each iteration including quantum thermal disorder. While the self-consistent determination of interatomic force constants in the stochastic scheme is a powerful technique which allows sampling of the phase space while including quantum thermal disorder at low temperatures, it does however requires the crystal under scrutiny to be in a stable configuration (i.e., without imaginary phonon modes). Such a priori knowledge can be obtained from experiments, but when data fall short it is unlikely to asses the stability of a system correctly.
(iii) Via sampling the potential energy surface using a machine-trained potential through molecular dynamics simulations with and without quantum nuclear effects (referred to as TDEP (MTP+CMD (+QTB))).

The anharmonicity measure defined by Knoop et al. was used to assess the degree of anharmonicity in the selected materials.^44^

### Machine Learning

On top of the obtained AIMD data sets, machine learning interatomic potentials were trained using the MLIP package (v2),^45^ which allows for moment tensor potentials (MTPs) to be constructed. The initial data sets contained AIMD simulations conducted at 100 and 200 K in the NVT and 300 K in the NpT ensemble for bulk ice Ih; 25, 100, and 200 K in the NVT, 300 K in the NpT ensemble for bulk ice XI; 25 K in the NVT ensemble for the (100):t0 1-layer and 2-layers, (110):t3 2-layers surfaces of ice Ih; and 25 K, 100 K, 200 K, 273 K, and 300 K for the (100):t0 2-layers and 25 K for the (110):t0 2-layers surfaces of ice XI.

Since the AIMD trajectories are correlated within short time periods, only every 10th step of the original trajectories was included in the respective initial training set. Next, MTPs were parametrized to describe the interatomic interactions. For computational efficiency, MTPs were first trained over subsampled AIMD trajectories. After the preliminary training of MTPs, the accuracy of the trained potentials was evaluated over the full AIMD trajectories and the configurations with high extrapolations grades are identified.^46^ Such selected configurations were then added to the original training sets, and the final MTPs were developed by retraining clean potentials over the updated training sets. We have used the default weights that express the importance of energies, atomic forces, and stresses in optimizing MTPs, i.e., they were set to 1, 0.1, and 0.001, respectively. The MLIP_PHONOPY code was used to assess the phonon spectra directly from the trained MTPs.^47^

### Molecular Dynamics

After the MPTs were trained, they were used in classical molecular dynamics (CMD) simulations, which in this work were conducted with the LAMMPS package.^48,49^ There, a Nosé–Hoover thermostat (NVT ensemble) was used with a time step of 1 fs, which subsequently was used to equilibrate the structures for a total of 1 ns before a trajectory of 1 ns was sampled taking every 1000th time step for sampling and thermodynamic analysis. From there, forces were extracted for subsequent TDEP calculations. To incorporate nuclear quantum effects, we employ the quantum thermal bath (QTB) technique proposed by Dammak and co-workers^50^ and implemented by Shen and Reed^51^ in the LAMMPS package by utilizing the QBMSST algorithm by Barrat et al.^52^

### Elastic Properties

Elastic constants, that characterize the stiffness of a material, have been obtained with LAMMPS in two ways: (i) at zero temperature, by deforming the simulation box and measuring the change in the stress tensor, and (ii) at finite temperature, by measuring the change in the average stress tensor in an NVT simulations where the cell undergoes finite deformation.^53^ A review of the advantages and disadvantages of all of these methods is provided in the work of Clavier et al.^54^

### Spectra

The temperature-dependent first-order Raman spectrum was computed with TDEP as free accessible in the code repository (https://github.com/tdep-developers/tdep-tutorials/tree/main/07_Raman). The Raman tensor is obtained from the changes in the susceptibility with respect to mode displacements. The calculation ingredients are the second and third order force constants, the dielectric tensor of each displaced geometry, all coupled together with the spectral functions obtained for different incident wavevectors. An efficient way to obtain those has been implemented within the TDEP Tools package by Florian Knoop (https://github.com/flokno/tools.tdep).

## Results and Discussion

### Bulk Harmonic Phonons

**Figure 1:**
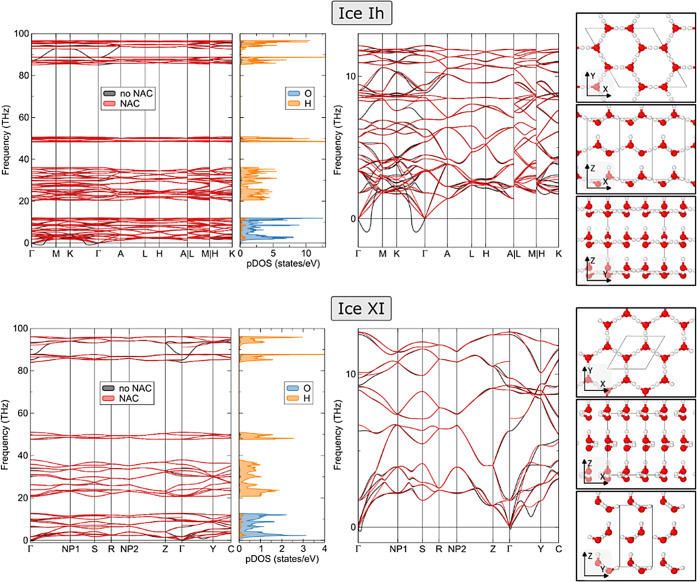
Phonon dispersion band structure together with atom-resolved phonon densities of states (left), a zoom in of the low-frequency region of the phonon dispersion (middle), and the crystallographic structure of the corresponding compounds (right). Reported values obtained using PBE-D2 together with phonopy (in the harmonic approximation) with red spheres representing oxygen and white spheres representing hydrogen.

The phonon dispersion relations of Ice in the Ih and XI phases along high-symmetry points in the reciprocal space are shown in Figure 1. The four distinct vibrational groups of modes (lattice, libration, bending, and stretching) are well reproduced, although there were no experimental results present in the literature that cover the whole range of phonon frequencies for comparison (to the best of our knowledge). The computed low energy phonons match reasonably well with available inelastic neutron scattering experiments^55,56^ (shown in Figure S1). It should also be noted that it is a challenging task to find direct one-to-one correspondence between the experimental setup, sample purity, measurement temperature, and pressure on one hand, with the defect-free, unstrained lattice employed in the theoretical model on the other side. The computed phononic densities of states (DOS) reveal dominant vibrational modes involving oxygen atoms in an energy range of up to 15 THz, while the remaining modes (with frequencies up to 100 THz) primarily arise from hydrogen atoms.

For any crystal structure to be stable, it needs to satisfy a set of conditions.^57,58^ One of the stability requirements of a crystal lattice is its invariability toward any small displacements of atoms. This condition requires that all phonon frequencies possess positive values. Ice Ih is computed to exhibit a fundamental phonon with a negative frequency, which renders the structure dynamically unstable. Careful tightening of computational parameters (exhausting possible sources of instability such as those listed in ref (59)) does not offer any remedy to the imaginary phonon. Furthermore, to rule out the effect of a particular technical implementation of DFT, we computed the phonon dispersion curves within a localized basis set (GTOs within CRYSTAL), which confirmed the presence of the imaginary phonon mode within Ice Ih. The same behavior was observed with a nonlocal van der Waals (vdW) functional (vdW-DF developed by Dion et al.^60^) and the small displacements method.

Given the disordered nature of hydrogen atoms in ice Ih (proton disorder), we argue that it is unlikely to expect static DFT calculations on a single reference simulation cell (and within the harmonic approximation) to capture the material-specific physics needed to reproduce the correct lattice dynamics behavior. One approach to capture the random nature of hydrogen is to employ a large cell, such as those developed by Hayward and Reimers,^61^ which however results in a drastically increased computational cost and consequently hardly allows us to step away from the harmonic approximation.

Before moving to an improved phonon description, we focus briefly on ice XI. Below approximately 200 K, hydrogen atoms in ice arrange themselves in periodic units, rendering the system proton ordered. Given that the XI phase is thermodynamically favorable in the low temperature regime, it could be argued that the imaginary phonon of the Ih phase indicates precisely that preference of the system to crystallize in the *Cmc*2_1_ symmetry. However, this does not appear to be the case, as the phonon dispersion curves of ice XI exhibit a similar imaginary phonon, albeit smaller in magnitude. The imaginary frequency was consistently reproduced by the CP2K/Phonopy approach, which confirmed the reliability of our calculations.

We also note in passing that for both phases the inclusion of the nonanalytical term to the dynamical matrix (still in the harmonic approximation) does not only reproduce the splitting between the phonon frequencies of optical modes but appears to stabilize the acoustic modes as well. As a result, the imaginary modes disappear. It remains to be explored whether this is a feature of the physics of the ice system or a numerical artifact.

### TDEP on Bulk Phases

To move beyond the harmonic approximation at zero Kelvin, we employ the TDEP method, which uses ab initio molecular dynamics simulation as a starting point for obtaining a (harmonic or higher order) potential energy surface at finite temperatures for subsequent lattice dynamics. Albeit not directly anharmonic, TDEP provides the most accurate harmonic approximation to the fully anharmonic energy landscape, thus encapsulating implicit information about anharmonicity via the temperature-dependent vibrational frequencies.^39^

**Figure 2:**
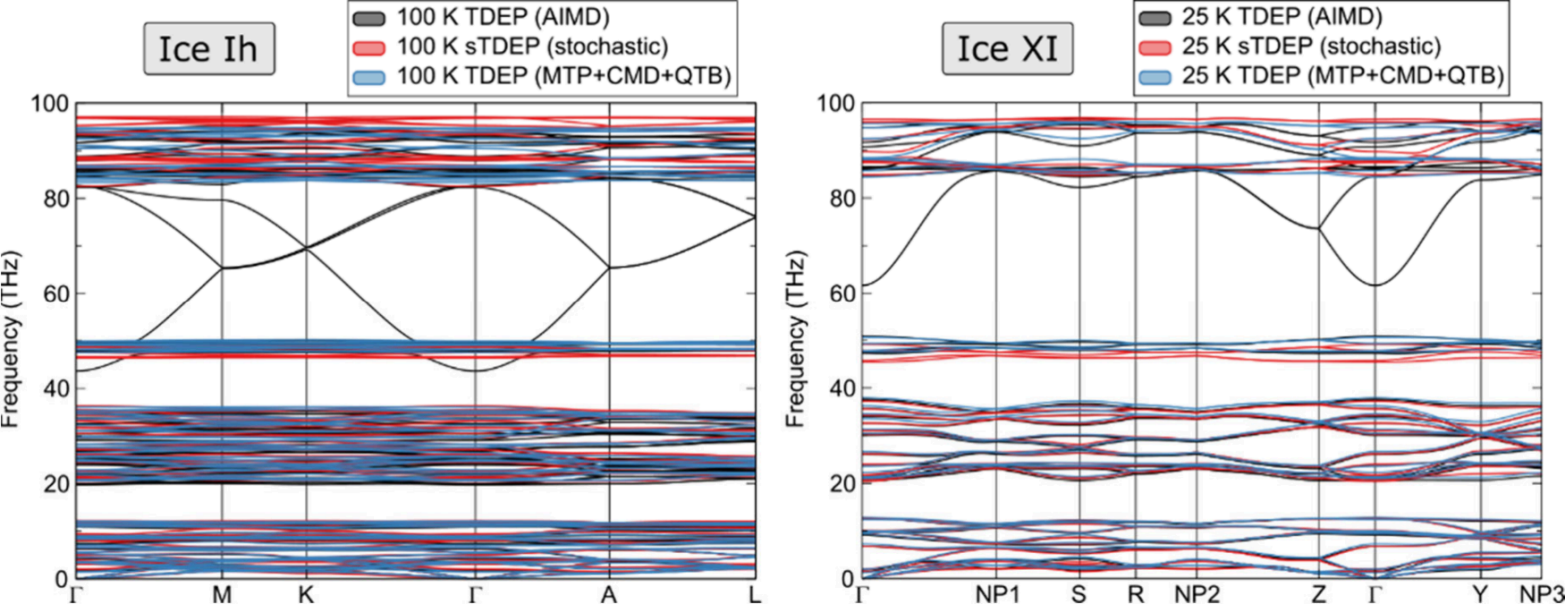
Calculated phonon dispersion bands using TDEP with three different ways of sampling the phase space of respective ice Ih and XI bulk phases: directly using ab initio molecular dynamics (AIMD), sampling uncorrelated supercells within a canonical ensemble (sTDEP), and by using classical molecular dynamics coupled to a quantum thermal bath (CMD+QTB).

The calculated dispersion relations at 100 K for Ice Ih and 25 K for ice XI are shown in Figure 2 (with a zoom in the low frequency region shown in Figure S2). It is immediately clear that the fundamental low energy phonons are all positive, thus implying stabilization of the respective phases when compared to the harmonic phonons. This also rules out the effect of nonanalytical corrections observed in the harmonic approximation, which is then most likely an artifact of adding the nonanalytical term to the dynamical matrix for harmonic phonons.

However, the high frequency phonons are computed to disperse across a large range of frequencies (so-called “*bubbles*”). These are the O–H stretching vibrations, which are known to exhibit strong nuclear quantum effects (NQEs) as a result of the low proton mass. NQEs play an important role in the vibrational spectra of aqueous systems and need to be incorporated carefully in addition to the anharmonicity of the O–H bond in the simulated potential energy surface.

To circumvent the short time lengths commonly accessible via AIMD trajectories (as a result of high resource demand), we move to trained MTPs and CMD. To assess the accuracy of the trained MTPs, we first perform phonon calculations in the harmonic regime using the trained potentials and compare those to earlier DFT values, with the results shown in Figure S2. It is clear that the MTP-based phonon dispersion bands excellently reproduce the ground state ones obtained from finite-different total energy DFT simulations, even when only passively trained on the initial training set. It is also appealing finding that the MTP-based phonons are fully stable (all frequencies found positive), as a results of the interatomic potential capturing the disordered nature of the ice system in an average fashion.

Further, to rule out the effect of trajectory length and corresponding correlation between time steps, we employed the MTPs trained on the AIMD trajectories and have performed a 1 ns CMD production run, from which we have extracted the phonon dispersion curves via TDEP. The resulting phonon bands displayed the same high frequency behavior, thus confirming the need for incorporating NQEs, especially at low temperatures.

There is a range of techniques available to include NQEs, such as path-integral molecular dynamics (PIMD),^62−64^ stochastic self-consistent harmonic approximation (SSCHA),^65^ self-consistent ab initio lattice dynamics (SCAILD),^66,67^ TDEP generalized to a full temperature-dependent potential (FTDP),^68^ TDEP utilizing stochastic sampling of the potential energy surfaces (sTDEP),^43^ or by coupling quantum statistics directly into molecular dynamics via a quantum thermal bath (QTB).^50,51^ We tested the latter two approaches within our workflow on ice Ih and ice XI and the results are shown in Figure 2.

The sTDEP sampling was found to converge the phonon dispersion spectrum already after less than a dozen iterations, fully localizing the O–H stretching vibrations in the region between 80 and 100 THz. While this is an efficient approach of generating configurations for investigating phonon and thermal properties, it relies on the assumption that the crystal is in a stable configuration. For the bulk phases of ice Ih and XI that requirement is known beforehand; however, this cannot be guaranteed for the thin film structures discussed in the upcoming paragraphs or systems where the stability is not known a priori.

Supplying standard MD with a quantum bath which includes energy quantization effects is found to overcome there limitation. As shown in Figure 2, the CMD+QTB approach and sTDEP approach result in the same result, even when the sTDEP sampling is not fully converged (quantified with a lower than necessary R2 value, listed in Table 1).

**Table 1:** Computed Degree of Anharmonicity (σ^A^) for Ice Ih and Ice XI in Their Bulk Phases Using Different Sampling Techniques to Explore the Phase Space^a^

		TDEP (AIMD)			sTDEP			TDEP (MTP+CMD)		
Phase	Temp. (K)	rc2 (Å)	R^2^	σ^A^	rc2 (Å)	R^2^	σ^A^	rc2 (Å)	R^2^	σ^A^
Ice Ih	25	-	-	-	-	-	-	5.5	0.98	0.15
	100	5.5	0.91	0.30	5.5	0.75	0.49	5.5	0.92	0.27
	200	5.0	0.81	-	-	-	-	5.5	0.85	0.39
	250	-	-	-	-	-	-	5.5	0.82	0.43
Ice XI	25	5.0	0.97	0.17	5.5	0.75	0.49	5.5	0.98	0.14
	100	-	-	-	-	-	-	5.5	0.92	0.28
	200	6.0	0.86	0.38	-	-	-	5.5	0.85	0.39
	250	-	-	-	-	-	-	5.5	0.81	0.44

a The following abbreviations are used: rc2, interaction cutoff for the second order interatomic force constants, and R^2^, measure of how well the obtained effective interatomic force constants describe the position–force data in a least-squares solution.

We have further assessed the level of anharmonicity in those two systems, employing the scheme proposed by Knoop et al.^44^ There, the degree of anharmonicity (σ^A^(T)) measures the standard deviation of the distributions of the anharmonic force components defined in the classical limit (neglecting quantum nuclear effects). The computed values for ice Ih and ice XI at various temperatures are given in Table 1. The two studied bulk systems show almost identical and non-negligible anharmonicity, which further increases significantly with temperature.

In summary of the bulk ice Ih and XI phases, we have identified a robust and computationally efficient approach (based on machine trained interatomic potentials coupled to a quantum thermal bath) to obtain the phonon properties of systems exhibiting significant anharmonicity and NQEs, regardless of their Debye temperature. The obvious limitations of these systems and approach is that the phonon picture breaks down in the classical sense once the phase transition into liquid water is approached.

### Surfaces and Phonons

We next focused on probing the phonon stability of well-defined slabs of ice Ih and XI, which we refer to as thin film structures in this work. This approach allows us to systematically create two-dimensional structures of ice in its two most stable phases, whereas the well-defined methodology provides a way of ruling out unstable structures before further analysis. Such filtering is of crucial importance for various reasons, for example, where the presence of imaginary frequencies in the phonon spectrum precludes the meaningful evaluation of thermodynamic properties. Ultimately, this screening is necessary to prevent subsequent waste of computational resources on compounds that are unphysical and at the same time scrutinize the reporting of novel phases of materials. At the same time, the included anharmonic and quantum nuclear effects can lead to phase stabilization, thus promoting compounds ruled-out purely on the basis of the harmonic approximation.

Ice Ih crystallizes in the hexagonal space group P6_3_cm (#185), resulting in four nonequivalent low-Miller index surfaces: (001), (100), (110), and (111). Cleaving the bulk along the (001) and (111) directions results in only dipolar terminations which would require reconstruction to remove the unphysical dipole, and we have therefore omitted from further analysis this in our first study due to the increased complexity of considering reconstructed thin films. Ice XI crystallizes in the orthorhombic space group Cmc2_1_ (#36), resulting in 7 nonequivalent low-Miller index surfaces, out of which the (001), (101), and (111) cuts are dipolar and again omitted from further analysis in this work. Given the potential confusion with nomenclature when cleaving ice XI arising from the (often unintended) use of a primitive versus conventional cell, we listed the corresponding Miller indices in Table S1. Within this work, we have used the Miller index nomenclature of the conventional unit cells.

The computed surface energies of the different terminations of one- and two-layer-thick slabs of ice Ih and XI in different terminations are listed in Table S2. The overall trend among the slabs shows that a low energy is required to cleave the bulk, which does not exceed 0.45 J/m^2^. Furthermore, to cleave a (100) surface of ice Ih (H-terminated) that is 1.25 nm thick requires as little as 0.17 J/m^2^. Thus, it is expected that such structures would easily form in systems containing water at low temperatures. However, without explicitly characterizing the vibrational properties, little can be concluded about the behavior of such systems at elevated temperatures or pressures by looking at the surface energy only.

In a first approximation, we directly employed the machine learned moment tensor potentials to probe the phonon dispersion relations of the cleaved slabs. That is, MTP has replaced DFT in the force evaluation step, while the final properties are evaluated with Phonopy. This approach has been shown to reproduce the phonon spectra and group velocities of complex 2D materials in good agreement with first-principles calculations.^69^ Although this approach is harmonic in nature, the MTP has been trained on the fully anharmonic potential energy surface; therefore, it remains to be assessed to what degree the total energies coming from MTPs contain such effects intrinsically.

We contrasted these results with phonon dispersion curves obtained with TDEP at a low temperature of 25 K from CMD calculations (+QTB where necessary) by using the trained MTPs. The crystal structures of the relevant thin films of ice Ih and XI, together with their corresponding phonon dispersion curves, are shown in Figure S4, Figure S5, and Figure S6.

Two stable structures of the ice Ih thin films were identified: the (100):t0 2-layers thick and (110):t0 2-layers thick slabs and one of the ice XI thin films, the (110):t0 2-layers thick geometry. Their phonons are found stable both within the finite-displacement method used with the trained MTPs on a static zero Kelvin simulation cell and within the TDEP method at 25 K, even when NQEs are omitted. For two additional thin film structures of ice XI, the (110):t0 1-layer thick and (010):t0 2-layers thick slabs, the MTP based phonons were found stable, while those obtained from the TDEP method demonstrated profound instabilities. This is an opposite case to the results of the bulk, where the TDEP based phonon dispersions were stabilized with temperature, anharmonicity, and NQEs. Here the simulation cell (within the harmonic approximation) captures a static snapshot that appears stable, while this geometry is not retained once the atoms are allowed to vibrate under the inclusion of all of the aforementioned effects.

**Figure 3:**
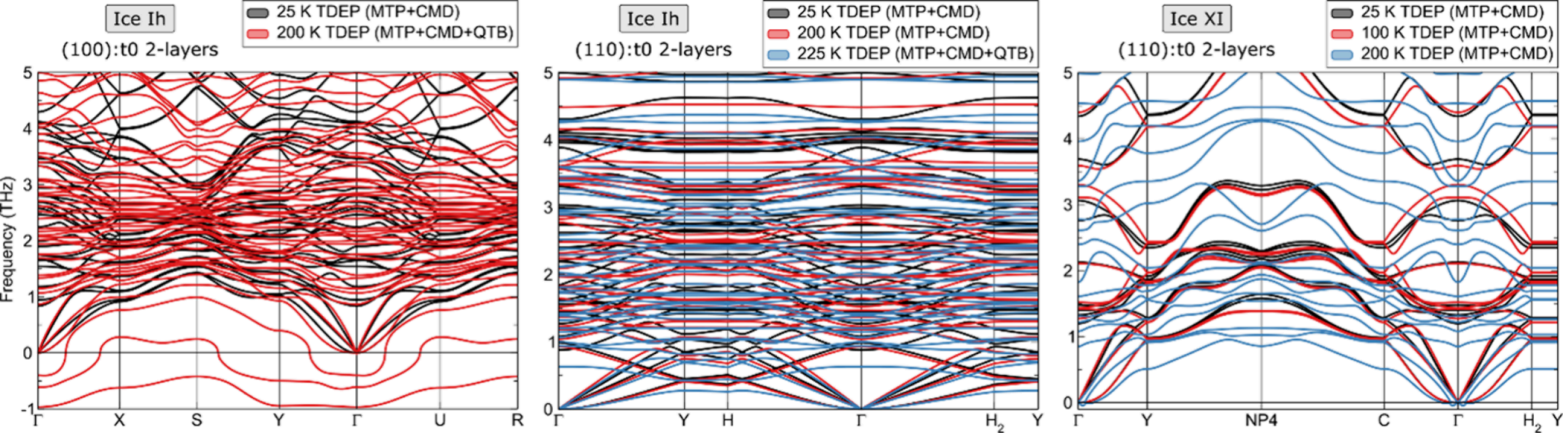
Calculated phonon dispersion bands of three identified stable ice Ih and XI thin film structure. Results were obtained using TDEP on top of a potential energy surface sampled with CMD(+QTB) and MTPs along different temperatures.

The calculated phonon dispersion curves for the three identified stable thin film structures of ices Ih and XI are shown in Figure 3. The characteristic parabolic shape of one acoustic mode appearing in 2D systems is well reproduced (a consequence of the broken periodicity in one direction), together with the two remaining acoustic modes which remain linear with respect to the momentum around Γ.

These three thin film structures, despite not having a substrate support, are found to show good stability up to at least 100 K, with the (110):t0 2-layers thin film of ice Ih (around 0.7 nm thick) remaining stable until 225 K and beyond. When probing temperatures around 250 K or higher, we start sampling the phase space near the phase transition region into liquid water, which leads to a breakdown of the phonon picture (i.e., long-range order or periodicity in the arrangement of atoms is progressively lost), and no further meaningful dispersion curves can be extracted.

We also note that for low temperature phonons of ice Ih and XI, NQEs play an important role in stabilizing the high frequency vibrations including hydrogen. However, with increasing temperature, they become crucial in stabilizing the low frequency phonons just as well. At temperatures above 200 K we noticed increasing convergence issues when employing classical MD only without supplying NQEs. On top of that, we calculated the anharmonicity degree for the three stable thin film structures, with results listed in Table 2. We note an increase of at least 50% in the intrinsic anharmonicity of the studied thin films when compared to their parent bulk structures. A trend of increasing anharmonicity with increasing temperature is also retained.

**Table 2:** Computed Degree of Anharmonicity (σ^A^) for the Stable Surfaces of Ice Ih and Ice XI Using Different Sampling Techniques to Explore the Phase Space^a^

			TDEP (AIMD)			TDEP (MTP+CMD)		
Phase	Surface	Temp. (K)	rc2 (Å)	R^2^	σ^A^	rc2 (Å)	R^2^	σ^A^
Ice Ih	(100):t0 2-layers	25 K	5.0	0.92	0.27	4.5	0.95	0.23
		200 K	-	-	-	4.5	0.54	0.67
	(110):t0 2-layers	25 K	-	-	-	5.0	0.93	0.27
		200 K	-	-	-	5.0	0.61	0.62
Ice XI	(110):t0 2-layers	25 K	6.0	0.87	0.35	5.5	0.90	0.31
		100 K	-	-	-	5.5	0.75	0.50
		200 K	-	-	-	6.5	0.44	0.75

a The following abbreviations are used: rc2, interaction cutoff for the second order interatomic force constants, and R^2^, measure of how well the obtained effective interatomic force constants describe the position–force data in a least-squares solution.

### Raman Spectrum

**Figure 4:**
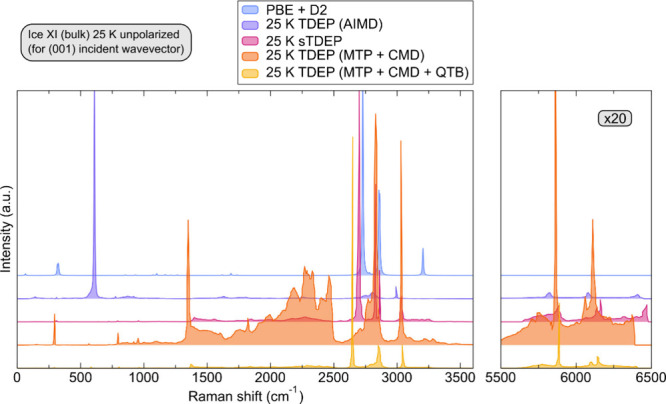
Comparison of the ice XI Raman spectrum at 25 K computed via several methods: DFT in harmonic approximation (PBE+D2), TDEP on potential energy sampled via ab initio molecular dynamics (TDEP(AIMD)), stochastic TDEP (sTDEP), and TDEP on potential energy sampled via classical molecular dynamics without (MTP+CMD) and with nuclear quantum effects (MTP+CMD+QTB). Values are reported for the (001) incident wavevector; the region between 5500 and 6500 cm^–1^ has been magnified for clarity, and spectra have been shifted along the *y*-axis for easier identification.

We also studied the temperature dependence of the Raman spectrum of ice, with the computed spectra of the XI phase shown in Figure 4 (no polarization). It is observed that the Raman spectrum obtained from the TDEP second and third order force constants sampled from AIMD is entirely inadequate in capturing the correct vibrational behavior of ice XI in the entire frequency range. The most prominent vibrations involving O–H stretching modes are severely underestimated, and the most active modes are assigned to be the libration ones at 600 cm^–1^. Subsequently, by simply extending the sampling of the potential energy surface (with only taking into account anharmonicity) to longer simulation times does not provide a satisfactory remedy of the outlined shortcomings. Albeit the most intense peaks are correctly assigned to the O–H stretching vibrations, the activity and intensity of the libration and bending modes in the range from 1300 to 2500 cm^–1^ are drastically overestimated. Both effects are correctly captured when NQEs are explicitly incorporated, which is the case within stochastic TDEP (sTDEP) and TDEP performed on top of a potential energy surface sampled with MTP + CMD + QTB. The prominent activity of the O–H modes in the region around 3000 cm^–1^ is correctly captured when comparing both their intensities with respect to each other as well as separation between modes. From here, it is easily concluded that in order to reproduce the Raman spectrum of ice, it is of paramount importance to introduce both anharmonic and quantum nuclear effects simultaneously into the description of the potential energy sampling scheme.

**Figure 5:**
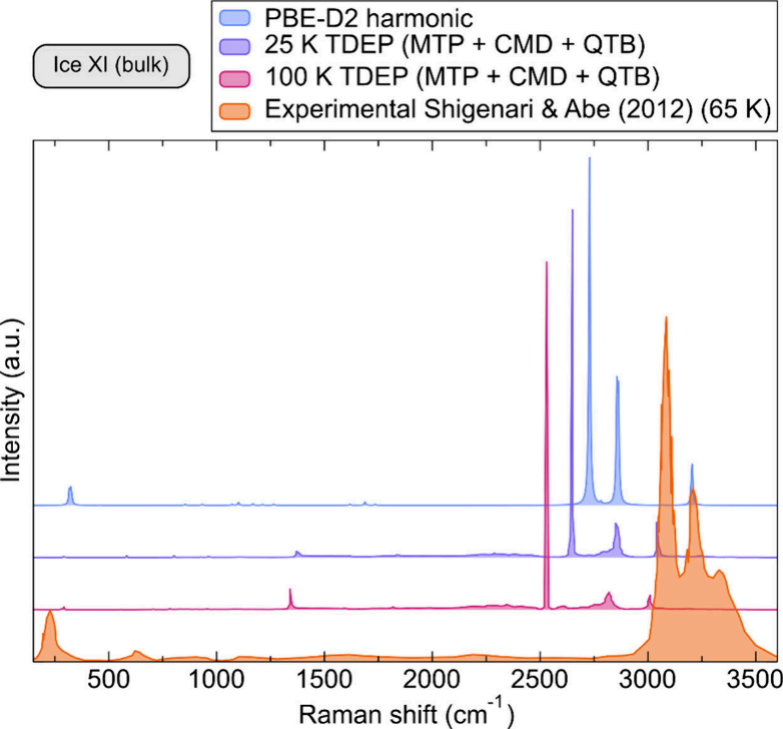
Computed Raman spectrum of bulk ice XI at different temperatures in comparison with different approximations (classic DFT with a semilocal functional, TDEP on top of CMD+QTB sampling using MTPs) with respect to experimentally available data from Shigenari and Abe.^70^ Values are reported for (001) incident wavevector, and the spectra have been shifted along the *y*-axis for easier comparison.

The comparison of the calculated spectra with experimentally available values from Shigenari and Abe^70^ is shown in Figure 5. Overall, the calculated frequencies underestimate the measured ones, which should not be a surprise given that the backbone of the simulations lies in a semilocal exchange-correlation functional, which is known for its underperformance in such systems.^71^ A straightforward remedy for this would be the inclusion of a hybrid functional, known to perform well in describing absolute values of computed frequencies, upon anharmonic corrections.^71−73^ A further peculiarity of the ice systems is the appearance of overtones in the region of around 6000 cm^–1^. Albeit being an order of magnitude smaller in intensity, they naturally appear as a result of the corrected anharmonic and quantum nuclear treatment. They however might be hardly detectable in experiments precisely due to their weak vibrational footprint.

To probe for eventual orientation effects, we computed the Raman spectrum of ice XI with respect to different incident wavevectors when approaching the Γ-point zone center, this time at a temperature of 100 K (shown in Figure S7). The variation of the intensities between different q-directions is minimal and for all three probed directions the inclusion of NQEs remains a critical point in order to capture the vibrational properties of ice XI correctly. Further, we computed the Raman spectrum of ice Ih at 100 K and probed polarization effects for the (001) incident wavevector, as shown in Figure S8, comparing once more sTDEP with TDEP(MTP+CMD+QTB). A strong split between the mode activities with respect to the polarization is observed, with the libration and bending modes dominantly active parallel to the incident laser and only the two O–H stretching modes, with the highest frequency being equally active for both polarizations. This perfectly reflects the symmetry of the system, with a large population of the O–H bonds directed along the *z*-axis of the crystal.

Finally, we compare the Raman spectrum of an exemplar thin film structure, specifically the (110):t0 2-layers thin surface of ice XI. The computed spectra are shown in Figures S9 and Figure S10. The former shows a comparison between spectra when NQEs are omitted and included, while the latter outlines differences between the Raman spectra of the bulk and the surface structures. It is straightforwardly observed how the NQEs play a large role in correctly describing the vibrational properties of 2D structures, more so than in the bulk. This effect is largely unaltered by the wavevector direction, with the spectra where NQEs are excluded overestimating the mode intensities over the whole frequency range.

From the contrast of the computed Raman spectra between the bulk and surface structures, several phenomena are observed. The most intense vibrations, originating from the hydrogen bearing stretching modes, are shifted to lower frequencies compared to their bulk counterpart. The libration and bending vibrations are found with a somewhat higher intensity than that in the bulk. And there appear novel high-frequency modes at 3000 cm^–1^ and greater. Their position depends on the incident wavevector and is an imprint of the reduced dimensionality of the surface structure differentiated from the corresponding bulk.

### Elastic Constants

To additionally confirm the stability of the outlined compounds and further test the trained MTPs as well as test whether the proposed workflow holds for such simulations, we calculated the elastic constants of ice Ih and XI. Given that elastic constants computed directly from TDEP are very sensitive to the convergence of the interatomic force constants, size of the simulation cell, or utilized cutoff,^74^ we have opted for computing the elastic properties directly from CMD simulations. The ground state as well as temperature dependent elastic constants of ice Ih and XI are listed in Tables S3 and S4.

The ground state elastic constants of ice Ih and XI are systematically overestimated when compared to available experimental data, albeit the correct trend among them is captured. All satisfy the mechanical stability criterion on top. This becomes an even more important criterion for 2D structures, where in addition to the dynamical stability (phonons), the Born–Huang mechanical stability criterion should be met.^75,76^ The calculated values for exemplar surfaces of ice Ih are given in Table S5. In the ground state, the (110):t3 1-layer surface can be immediately ruled out due to its vanishing elastic constants (which is not a surprise, given the disrupted structure), while other structures are found mechanically stable. With increasing temperature, the (100):t0 1-layer surface shown negative elastic constants, thus being mechanically unstable. All four other surfaces do not indicate any mechanical instabilities, yet for (110):t0 1-layer and (110):t3 2-layers the dynamical stability criterion is not satisfied as outlined earlier.

As such, these results further confirm the required synergy between the dynamic and mechanical stability criteria for geometrically stable structures. A word of caution is, however, required: to calculate elastic constants at finite temperature is notoriously challenging, as time averaging of different properties is required. As such, we have not intended to fully converge the given values but used this case as a proof of concept that our outlined chain of connected methods can be successfully extended to elastic properties as well. More importantly, the reported values contain nuclear quantum effects explicitly, not just in the phonon picture but also in the mechanical properties of the studies systems. This is achieved at a minimal increase in computational cost, compared to neglecting NQEs, which is something yet to be achieved with other methods that capture NQEs, to the best of the authors’ knowledge.

### Limitations and Outlook

Despite the explicit effort to include temperature, anharmonicity, or NQEs in our study of the bulk and thin film phases of ice, a couple of effects remained left out. The obvious one is pressure, which is known to shift the transition points in the phase diagram of water between the solid and liquid phases. Furthermore, we still need to establish the effect of a substrate on the identified stable thin films, as confined water has been shown to exhibit unusual properties compared to the bulk. Computationally, the choice of the exchange-correlation functional poses a hard limit on the data used not only for the harmonic phonons but moreover for the training of the interatomic potentials. However, despite all of the outlined deficiencies, the models presented here are robust and offer an efficient way of including a range of important phenomena, particularly when studying low temperature systems. Moreover, we have demonstrated not only its applicability to systems of perfect 3D structures but also that 2D thin film structures can be treated at the same footing with minimal cost increased.

## Conclusion

In this comprehensive study, we investigated the vibrational properties of ice Ih and ice XI, focusing on both bulk and thin film structures. Employing a chain of connecting computational techniques, including density functional theory (DFT), machine learning interatomic potentials (MTPs), and temperature-dependent effective potential (TDEP) methods, we elucidated the complex interplay between anharmonicity, nuclear quantum effects (NQEs), and temperature on the phonon spectra of these ice phases. Our investigation has revealed a number of intriguing phenomena, such as the presence of imaginary phonon modes in both bulk phases, indicating their dynamical instability, the stabilization of phonons with temperature, and the importance of NQEs when sampling the phase space region of hydrogen stretching vibrations. Furthermore, we identified at least three stable thin film structures and quantified their anharmonicity, shedding light on their stability and phonon characteristics. Our analysis of the Raman spectrum highlights the importance of accounting for anharmonicity and NQEs in accurately predicting vibrational properties. Additionally, our simulation strategy reveals a possibility of obtaining accuracy equivalent to that from stochastic TDEP, yet with lifting the necessary condition of a system having no imaginary frequencies in its phonon spectrum.

Overall, our findings underscore the significance of combining advanced computational methodologies when studying the intricate vibrational dynamics of crystalline ice phases (regardless of their dimensionality, not limited to only those compounds by any means) and paving the way for deeper insights into their thermodynamic and structural properties. In addition, we suggest that utilizing a workflow consisting of TDEP to obtain force constants from MTPs and CMD with additional nuclear quantum effects (by using QTB) provides a favorable trade-off between accuracy and speed to model ice in its both bulk and thin film forms.

## Supporting Information Available

Calculated phonon dispersion of ice Ih in comparison with experimentally available data, calculated phonon dispersion of ice XI comparing DFT and MTPs, calculated phonon dispersion band using TDEP and three different samplings, calculated surface energies of ice Ih and ice XI, atomic structure of thin films of ice Ih and ice XI together with their respective phonon dispersion curves, calculated Raman spectra of bulk ice XI in comparison with and without nuclear quantum effects, calculated Raman spectra of ice Ih comparing different potential energy sampling techniques, calculated Raman spectra of ice XI thin film surfaces contrasting different polarizations as well as showing effect of including and omitting nuclear quantum effects, and computed elastic constants of ice Ih and ice XI (PDF)

## References

[ref1] Vincendon M.; Forget F.; Mustard J. Water Ice at Low to Midlatitudes on Mars. J. Geophys. Res. Planets 2010, 115 (E10), E10001 10.1029/2010JE003584.

[ref2] Wang D.; Tian Y.; Jiang L. Abnormal Properties of Low-Dimensional Confined Water. Small 2021, 17 (31), 2100788 10.1002/smll.202100788. 34176214

[ref3] Kimmel G. A.; Matthiesen J.; Baer M.; Mundy C. J.; Petrik N. G.; Smith R. S.; Dohnálek Z.; Kay B. D. No Confinement Needed: Observation of a Metastable Hydrophobic Wetting Two-Layer Ice on Graphene. J. Am. Chem. Soc. 2009, 131 (35), 12838–12844. 10.1021/ja904708f. 19670866

[ref4] Zhang P.; Tian L.; Zhang Z. P.; Shao G.; Li J. C. Investigation of the Hydrogen Bonding in Ice Ih by First-Principles Density Function Methods. J. Chem. Phys. 2012, 137 (4), 044504 10.1063/1.4736853. 22852628

[ref5] Shi L.; Skinner J. L. Proton Disorder in Ice Ih and Inhomogeneous Broadening in Two-Dimensional Infrared Spectroscopy. J. Phys. Chem. B 2013, 117 (49), 15536–15544. 10.1021/jp405860u. 23937451

[ref6] Tribello G. A.; Slater B. Proton Ordering Energetics in Ice Phases. Chem. Phys. Lett. 2006, 425 (4–6), 246–250. 10.1016/j.cplett.2006.04.111.

[ref7] Schönherr M.; Slater B.; Hutter J.; Vandevondele J. Dielectric Properties of Water Ice, the Ice Ih/Xi Phase Transition, and an Assessment of Density Functional Theory. J. Phys. Chem. B 2014, 118 (2), 590–596. 10.1021/jp4103355. 24392971

[ref8] Pan D.; Liu L. M.; Tribello G. A.; Slater B.; Michaelides A.; Wang E. Surface Energy and Surface Proton Order of the Ice Ih Basal and Prism Surfaces. J. Phys.: Condens. Matter 2010, 22 (7), 074209 10.1088/0953-8984/22/7/074209. 21386387

[ref9] Hamada I. A van Der Waals Density Functional Study of Ice Ih. J. Chem. Phys. 2010, 133 (21), 214503 10.1063/1.3507916. 21142304

[ref10] Zhao W. H.; Wang L.; Bai J.; Yuan L. F.; Yang J.; Zeng X. C. Highly Confined Water: Two-Dimensional Ice, Amorphous Ice, and Clathrate Hydrates. Acc. Chem. Res. 2014, 47 (8), 2505–2513. 10.1021/ar5001549. 25088018

[ref11] Morawietz T.; Singraber A.; Dellago C.; Behler J. How van Der Waals Interactions Determine the Unique Properties of Water. Proc. Natl. Acad. Sci. U. S. A. 2016, 113 (30), 8368–8373. 10.1073/pnas.1602375113. 27402761 PMC4968748

[ref12] Slater B.; Michaelides A. Surface Premelting of Water Ice. Nat. Rev. Chem. 2019, 3 (3), 172–188. 10.1038/s41570-019-0080-8.

[ref13] Fumagalli L.; Esfandiar A.; Fabregas R.; Hu S.; Ares P.; Janardanan A.; Yang Q.; Radha B.; Taniguchi T.; Watanabe K.; Gomila G.; Novoselov K. S.; Geim A. K. Anomalously Low Dielectric Constant of Confined Water. Science (80-.). 2018, 360 (6395), 1339–1342. 10.1126/science.aat4191. 29930134

[ref14] Artemov V. G. A Unified Mechanism for Ice and Water Electrical Conductivity from Direct Current to Terahertz. Phys. Chem. Chem. Phys. 2019, 21 (15), 8067–8072. 10.1039/C9CP00257J. 30932107

[ref15] Kresse G.; Joubert D. From Ultrasoft Pseudopotentials to the Projector Augmented-Wave Method. Phys. Rev. B 1999, 59 (3), 1758–1775. 10.1103/PhysRevB.59.1758.

[ref16] Kresse G.; Furthmüller J. Efficiency of Ab-Initio Total Energy Calculations for Metals and Semiconductors Using a Plane-Wave Basis Set. Comput. Mater. Sci. 1996, 6 (1), 15–50. 10.1016/0927-0256(96)00008-0.

[ref17] Kresse G.; Furthmüller J. Efficient Iterative Schemes for Ab Initio Total-Energy Calculations Using a Plane-Wave Basis Set. Phys. Rev. B 1996, 54 (16), 11169–11186. 10.1103/PhysRevB.54.11169. 9984901

[ref18] Perdew J. P.; Burke K.; Ernzerhof M. Generalized Gradient Approximation Made Simple. Phys. Rev. Lett. 1996, 77 (18), 3865–3868. 10.1103/PhysRevLett.77.3865. 10062328

[ref19] Grimme S. Semiempirical GGA-Type Density Functional Constructed with a Long-Range Dispersion Correction. J. Comput. Chem. 2006, 27 (15), 1787–1799. 10.1002/jcc.20495. 16955487

[ref20] Monkhorst H. J.; Pack J. D. Special Points for Brillouin-Zone Integrations. Phys. Rev. B 1976, 13 (12), 5188–5192. 10.1103/PhysRevB.13.5188.

[ref21] Togo A.; Chaput L.; Tanaka I.; Hug G. First-Principles Phonon Calculations of Thermal Expansion in Ti_3_SiC_2_, Ti_3_AlC_2_, and Ti_3_GeC_2_. Phys. Rev. B 2010, 81 (17), 174301 10.1103/PhysRevB.81.174301.

[ref22] Togo A.; Tanaka I. First Principles Phonon Calculations in Materials Science. Scr. Mater. 2015, 108, 1–5. 10.1016/j.scriptamat.2015.07.021.

[ref23] Skelton J. M.; Parker S. C.; Togo A.; Tanaka I.; Walsh A. Thermal Physics of the Lead Chalcogenides PbS, PbSe, and PbTe from First Principles. Phys. Rev. B 2014, 89 (20), 205203 10.1103/PhysRevB.89.205203.

[ref24] Gonze X.; Lee C. Dynamical Matrices, Born Effective Charges, Dielectric Permittivity Tensors, and Interatomic Force Constants from Density-Functional Perturbation Theory. Phys. Rev. B - Condens. Matter Mater. Phys. 1997, 55 (16), 10355–10368. 10.1103/PhysRevB.55.10355.

[ref25] Kühne T. D.; Iannuzzi M.; Del Ben M.; Rybkin V. V.; Seewald P.; Stein F.; Laino T.; Khaliullin R. Z.; Schütt O.; Schiffmann F.; Golze D.; Wilhelm J.; Chulkov S.; Bani-Hashemian M. H.; Weber V.; Borštnik U.; Taillefumier M.; Jakobovits A. S.; Lazzaro A.; Pabst H.; Müller T.; Schade R.; Guidon M.; Andermatt S.; Holmberg N.; Schenter G. K.; Hehn A.; Bussy A.; Belleflamme F.; Tabacchi G.; Glöß A.; Lass M.; Bethune I.; Mundy C. J.; Plessl C.; Watkins M.; VandeVondele J.; Krack M.; Hutter J. CP2K: An Electronic Structure and Molecular Dynamics Software Package -Quickstep: Efficient and Accurate Electronic Structure Calculations. J. Chem. Phys. 2020, 152 (19), 194103 10.1063/5.0007045. 33687235

[ref26] VandeVondele J.; Krack M.; Mohamed F.; Parrinello M.; Chassaing T.; Hutter J. Quickstep: Fast and Accurate Density Functional Calculations Using a Mixed Gaussian and Plane Waves Approach. Comput. Phys. Commun. 2005, 167 (2), 103–128. 10.1016/j.cpc.2004.12.014.

[ref27] VandeVondele J.; Hutter J. Gaussian Basis Sets for Accurate Calculations on Molecular Systems in Gas and Condensed Phases. J. Chem. Phys. 2007, 127 (11), 114105 10.1063/1.2770708. 17887826

[ref28] Krack M. Pseudopotentials for H to Kr Optimized for Gradient-Corrected Exchange-Correlation Functionals. Theor. Chem. Acc. 2005, 114 (1), 145–152. 10.1007/s00214-005-0655-y.

[ref29] Goedecker S.; Teter M.; Hutter J. Separable Dual-Space Gaussian Pseudopotentials. Phys. Rev. B 1996, 54 (3), 1703–1710. 10.1103/PhysRevB.54.1703. 9986014

[ref30] Hartwigsen C.; Goedecker S.; Hutter J. Relativistic Separable Dual-Space Gaussian Pseudopotentials from H to Rn. Phys. Rev. B 1998, 58 (7), 3641–3662. 10.1103/PhysRevB.58.3641. 9986014

[ref31] Dovesi R.; Saunders V. R.; Roetti C.; Orlando R.; Zicovich-Wilson C. M.; Pascale F.; Civalleri B.; Doll K.; Harrison N. M.; Bush I. J.; D’Arco P.; Llunell M.; Causà M.; Noël Y.; Maschio L.; Erba A.; Rerat M.; Casassa S. CRYSTAL17 User’s Manual; University of Torino: Torino, 2017.

[ref32] Dovesi R.; Erba A.; Orlando R.; Zicovich-Wilson C. M.; Civalleri B.; Maschio L.; Rérat M.; Casassa S.; Baima J.; Salustro S.; Kirtman B. Quantum-Mechanical Condensed Matter Simulations with CRYSTAL. Wiley Interdiscip. Rev. Comput. Mol. Sci. 2018, 8 (4), 1–36. 10.1002/wcms.1360.

[ref33] Becke A. D. A New Mixing of Hartree–Fock and Local Density-functional Theories. J. Chem. Phys. 1993, 98 (2), 1372–1377. 10.1063/1.464304.

[ref34] Lee C.; Yang W.; Parr R. G. Development of the Colle-Salvetti Correlation-Energy Formula into a Functional of the Electron Density. Phys. Rev. B 1988, 37 (2), 785–789. 10.1103/PhysRevB.37.785. 9944570

[ref35] Vilela Oliveira D.; Laun J.; Peintinger M. F.; Bredow T. BSSE-Correction Scheme for Consistent Gaussian Basis Sets of Double- and Triple-Zeta Valence with Polarization Quality for Solid-State Calculations. J. Comput. Chem. 2019, 40 (27), 2364–2376. 10.1002/jcc.26013. 31260123

[ref36] Maschio L.; Kirtman B.; Orlando R.; Rèrat M. Ab Initio Analytical Infrared Intensities for Periodic Systems through a Coupled Perturbed Hartree-Fock/Kohn-Sham Method. J. Chem. Phys. 2012, 137 (20), 204113 10.1063/1.4767438. 23205987

[ref37] Maschio L.; Kirtman B.; Rérat M.; Orlando R.; Dovesi R. Ab Initio Analytical Raman Intensities for Periodic Systems through a Coupled Perturbed Hartree-Fock/Kohn-Sham Method in an Atomic Orbital Basis. I. Theory. J. Chem. Phys. 2013, 139 (16), 164101 10.1063/1.4824442. 24181998

[ref38] Maschio L.; Kirtman B.; Rérat M.; Orlando R.; Dovesi R. Ab Initio Analytical Raman Intensities for Periodic Systems through a Coupled Perturbed Hartree-Fock/Kohn-Sham Method in an Atomic Orbital Basis. I. Theory. J. Chem. Phys. 2013, 139 (16), 164101 10.1063/1.4824442. 24181998

[ref39] Hellman O.; Abrikosov I. A.; Simak S. I. Lattice Dynamics of Anharmonic Solids from First Principles. Phys. Rev. B 2011, 84 (18), 180301 10.1103/PhysRevB.84.180301.

[ref40] Hellman O.; Steneteg P.; Abrikosov I. A.; Simak S. I. Temperature Dependent Effective Potential Method for Accurate Free Energy Calculations of Solids. Phys. Rev. B - Condens. Matter Mater. Phys. 2013, 87 (10), 1–8. 10.1103/PhysRevB.87.104111.

[ref41] Hellman O.; Abrikosov I. A. Temperature-Dependent Effective Third-Order Interatomic Force Constants from First Principles. Phys. Rev. B - Condens. Matter Mater. Phys. 2013, 88 (14), 1–5. 10.1103/PhysRevB.88.144301.

[ref42] Knoop F.; Shulumba N.; Castellano A.; Batista J. P. A.; Farris R.; Verstraete M. J.; Heine M.; Broido D.; Kim D. S.; Klarbring J.; Abrikosov I. A.; Simak S. I.; Hellman O. TDEP: Temperature Dependent Effective Potentials. J. Open Source Softw. 2024, 9 (94), 6150 10.21105/joss.06150.

[ref43] Shulumba N.; Hellman O.; Minnich A. J. Intrinsic Localized Mode and Low Thermal Conductivity of PbSe. Phys. Rev. B 2017, 95 (1), 1–9. 10.1103/PhysRevB.95.014302. 29219537

[ref44] Knoop F.; Purcell T. A. R.; Scheffler M.; Carbogno C. Anharmonicity Measure for Materials. Phys. Rev. Mater. 2020, 4 (8), 083809 10.1103/PhysRevMaterials.4.083809.

[ref45] Novikov I. S.; Gubaev K.; Podryabinkin E. V.; Shapeev A. V. The MLIP Package: Moment Tensor Potentials with MPI and Active Learning. Mach. Learn. Sci. Technol. 2021, 2 (2), 025002 10.1088/2632-2153/abc9fe.

[ref46] Podryabinkin E. V.; Shapeev A. V. Active Learning of Linearly Parametrized Interatomic Potentials. Comput. Mater. Sci. 2017, 140, 171–180. 10.1016/j.commatsci.2017.08.031.

[ref47] Mortazavi B.; Novikov I. S.; Podryabinkin E. V.; Roche S.; Rabczuk T.; Shapeev A. V.; Zhuang X. Exploring Phononic Properties of Two-Dimensional Materials Using Machine Learning Interatomic Potentials. Appl. Mater. Today 2020, 20, 100685 10.1016/j.apmt.2020.100685.

[ref48] Plimpton S. Fast Parallel Algorithms for Short-Range Molecular Dynamics. J. Comput. Phys. 1995, 117 (1), 1–19. 10.1006/jcph.1995.1039.

[ref49] Thompson A. P.; Aktulga H. M.; Berger R.; Bolintineanu D. S.; Brown W. M.; Crozier P. S.; in ’t Veld P. J.; Kohlmeyer A.; Moore S. G.; Nguyen T. D.; Shan R.; Stevens M. J.; Tranchida J.; Trott C.; Plimpton S. J. LAMMPS - a Flexible Simulation Tool for Particle-Based Materials Modeling at the Atomic, Meso, and Continuum Scales. Comput. Phys. Commun. 2022, 271, 108171 10.1016/j.cpc.2021.108171.

[ref50] Dammak H.; Chalopin Y.; Laroche M.; Hayoun M.; Greffet J. J. Quantum Thermal Bath for Molecular Dynamics Simulation. Phys. Rev. Lett. 2009, 103 (19), 1–4. 10.1103/PhysRevLett.103.190601. 20365913

[ref51] Shen Y.; Reed E. J. Quantum Nuclear Effects in Stishovite Crystallization in Shock-Compressed Fused Silica. J. Phys. Chem. C 2016, 120 (31), 17759–17766. 10.1021/acs.jpcc.6b05083.

[ref52] Barrat J.-L.; Rodney D. Portable Implementation of a Quantum Thermal Bath for Molecular Dynamics Simulations. J. Stat. Phys. 2011, 144 (3), 679–689. 10.1007/s10955-011-0193-z.

[ref53] Clavier G.; Thompson A. P. Computation of the Thermal Elastic Constants for Arbitrary Manybody Potentials in LAMMPS Using the Stress-Fluctuation Formalism. Comput. Phys. Commun. 2023, 286, 108674 10.1016/j.cpc.2023.108674.

[ref54] Clavier G.; Desbiens N.; Bourasseau E.; Lachet V.; Brusselle-Dupend N.; Rousseau B. Computation of Elastic Constants of Solids Using Molecular Simulation: Comparison of Constant Volume and Constant Pressure Ensemble Methods. Mol. Simul. 2017, 43 (17), 1413–1422. 10.1080/08927022.2017.1313418.

[ref55] Strässle T.; Saitta A. M.; Klotz S.; Braden M. Phonon Dispersion of Ice under Pressure. Phys. Rev. Lett. 2004, 93 (22), 1–4. 10.1103/PhysRevLett.93.225901. 15601100

[ref56] Dorner B. Inelastic Neutron Scattering from Ice and Other Proton-Containing Substances. J. Glaciol. 1978, 21 (85), 231–240. 10.3189/S0022143000033438.

[ref57] Baroni S.; De Gironcoli S.; Dal Corso A.; Giannozzi P. Phonons and Related Crystal Properties from Density-Functional Perturbation Theory. Rev. Mod. Phys. 2001, 73 (2), 515–562. 10.1103/RevModPhys.73.515.

[ref58] Lukačević I. High Pressure Lattice Dynamics, Dielectric and Thermodynamic Properties of SrO. Phys. B Condens. Matter 2011, 406 (18), 3410–3416. 10.1016/j.physb.2011.06.010.

[ref59] Pallikara I.; Kayastha P.; Skelton J. M.; Whalley L. D. The Physical Significance of Imaginary Phonon Modes in Crystals. Electron. Struct. 2022, 4 (3), 033002 10.1088/2516-1075/ac78b3.

[ref60] Dion M.; Rydberg H.; Schröder E.; Langreth D. C.; Lundqvist B. I. Van Der Waals Density Functional for General Geometries. Phys. Rev. Lett. 2004, 92 (24), 246401 10.1103/PhysRevLett.92.246401. 15245113

[ref61] Hayward J. A.; Reimers J. R. Unit Cells for the Simulation of Hexagonal Ice. J. Chem. Phys. 1997, 106 (4), 1518–1529. 10.1063/1.473300.

[ref62] Ceriotti M.; Fang W.; Kusalik P. G.; McKenzie R. H.; Michaelides A.; Morales M. A.; Markland T. E. Nuclear Quantum Effects in Water and Aqueous Systems: Experiment, Theory, and Current Challenges. Chem. Rev. 2016, 116 (13), 7529–7550. 10.1021/acs.chemrev.5b00674. 27049513

[ref63] Conte R.; Aieta C.; Botti G.; Cazzaniga M.; Gandolfi M.; Lanzi C.; Mandelli G.; Moscato D.; Ceotto M. Anharmonicity and Quantum Nuclear Effects in Theoretical Vibrational Spectroscopy: A Molecular Tale of Two Cities. Theor. Chem. Acc. 2023, 142 (5), 53 10.1007/s00214-023-02993-y.

[ref64] Eltareb A.; Lopez G. E.; Giovambattista N. The Importance of Nuclear Quantum Effects on the Thermodynamic and Structural Properties of Low-Density Amorphous Ice: A Comparison with Hexagonal Ice. J. Phys. Chem. B 2023, 127 (20), 4633–4645. 10.1021/acs.jpcb.3c01025. 37178124 PMC10329782

[ref65] Monacelli L.; Bianco R.; Cherubini M.; Calandra M.; Errea I.; Mauri F. The Stochastic Self-Consistent Harmonic Approximation: Calculating Vibrational Properties of Materials with Full Quantum and Anharmonic Effects. J. Phys.: Condens. Matter 2021, 33 (36), 363001 10.1088/1361-648X/ac066b. 34049302

[ref66] Souvatzis P.; Eriksson O.; Katsnelson M. I.; Rudin S. P. Entropy Driven Stabilization of Energetically Unstable Crystal Structures Explained from First Principles Theory. Phys. Rev. Lett. 2008, 100 (9), 95901 10.1103/PhysRevLett.100.095901. 18352725

[ref67] van Roekeghem A.; Carrete J.; Mingo N. Quantum Self-Consistent Ab-Initio Lattice Dynamics. Comput. Phys. Commun. 2021, 263, 107945 10.1016/j.cpc.2021.107945.

[ref68] Geng H. Y. Full Temperature-Dependent Potential and Anharmonicity in Metallic Hydrogen: Colossal NQE and the Consequences. J. Phys. Chem. C 2022, 126 (45), 19355–19366. 10.1021/acs.jpcc.2c05027.

[ref69] Mortazavi B.; Podryabinkin E. V.; Novikov I. S.; Roche S.; Rabczuk T.; Zhuang X.; Shapeev A. V. Efficient Machine-Learning Based Interatomic Potentialsfor Exploring Thermal Conductivity in Two-Dimensional Materials. J. Phys. Mater. 2020, 3 (2), 02LT02 10.1088/2515-7639/ab7cbb.

[ref70] Shigenari T.; Abe K. Vibrational Modes of Hydrogens in the Proton Ordered Phase XI of Ice: Raman Spectra above 400 Cm–1. J. Chem. Phys. 2012, 136 (17), 174504 10.1063/1.3702595. 22583246

[ref71] Erba A.; Maul J.; Ferrabone M.; Dovesi R.; Rérat M.; Carbonnière P. Anharmonic Vibrational States of Solids from DFT Calculations. Part II: Implementation of the VSCF and VCI Methods. J. Chem. Theory Comput. 2019, 15 (6), 3766–3777. 10.1021/acs.jctc.9b00294. 31038948

[ref72] Erba A.; Maul J.; Ferrabone M.; Carbonnière P.; Rérat M.; Dovesi R. Anharmonic Vibrational States of Solids from DFT Calculations. Part I: Description of the Potential Energy Surface. J. Chem. Theory Comput. 2019, 15 (6), 3755–3765. 10.1021/acs.jctc.9b00293. 31038943

[ref73] Schireman R. G.; Maul J.; Erba A.; Ruggiero M. T. Anharmonic Coupling of Stretching Vibrations in Ice: A Periodic VSCF and VCI Description. J. Chem. Theory Comput. 2022, 18 (7), 4428–4437. 10.1021/acs.jctc.2c00217. 35737003

[ref74] Shulumba N.; Hellman O.; Rogström L.; Raza Z.; Tasnádi F.; Abrikosov I. A.; Odén M. Temperature-Dependent Elastic Properties of Ti1–xAlxN Alloys. Appl. Phys. Lett. 2015, 107 (23), 231901 10.1063/1.4936896.

[ref75] Ghosal S.; Chowdhury S.; Jana D. Impressive Thermoelectric Figure of Merit in Two-Dimensional Tetragonal Pnictogens: A Combined First-Principles and Machine-Learning Approach. ACS Appl. Mater. Interfaces 2021, 13 (49), 59092–59103. 10.1021/acsami.1c18200. 34843210

[ref76] Wang H.; Li T.; Liu X.; Zhu W.; Chen Z.; Li Z.; Yang J. Mech2d: An Efficient Tool for High-Throughput Calculation of Mechanical Properties for Two-Dimensional Materials. Molecules 2023, 28, 4337 10.3390/molecules28114337. 37298813 PMC10254924

