# Supplementary material for: Water Is Cool: Advanced Phonon Dynamics in Ice Ih and Ice XI via Machine Learning Potentials and Quantum Nuclear Vibrations
Source: J Chem Theory Comput. 2025 Feb 7;21(4):1978–89. doi: 10.1021/acs.jctc.4c01582 (PMC11866926; doi:10.1021/acs.jctc.4c01582)
Supplement: Supplementary file 1 — ct4c01582_si_001.pdf [file ct4c01582_si_001.pdf]

Supplementary information file for

Water is Cool: Advanced Phonon Dynamics in Ice Ih and Ice XI via Machine Learning Potentials and Quantum Nuclear Vibrations

Aleksandar Živković[1,4], Umberto Terranova[2,*], Nora H. de Leeuw[1,3,*]

[1] Department of Earth Sciences, Utrecht University, Princetonlaan 8a, 3584CB Utrecht, The Netherlands
[2] Faculty of Medicine and Health Sciences, Crewe Campus, University of Buckingham, Crewe, CW1 5DU, UK
[3] School of Chemistry, University of Leeds, Leeds LS2 9JT, United Kingdom
[4] Institute for Inorganic Chemistry Christian-Albrechts-Universität zu Kiel, Max-Eyth-Str. 2, 24118 Kiel,Germany
[*] Corresponding authors: azivkovic@uu.nl, umberto.terranova@buckingham.ac.uk, n.h.deleeuw@leeds.ac.uk

Bulk behaviour

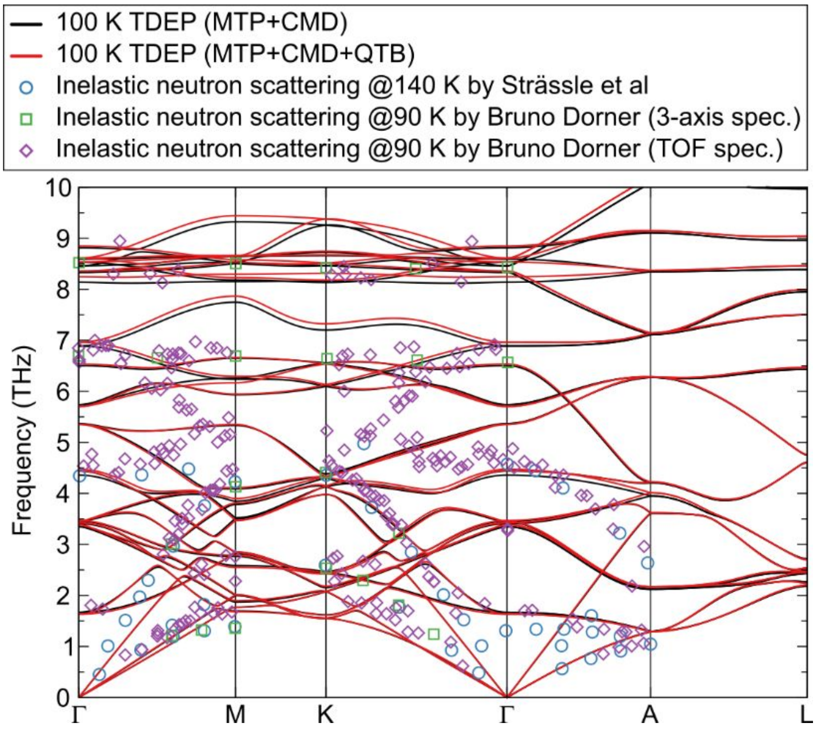

Figure S1. Comparison of computed phonon dispersions of ice Ih with available experimental literature data obtained by inelastic neutron scattering[1,2].

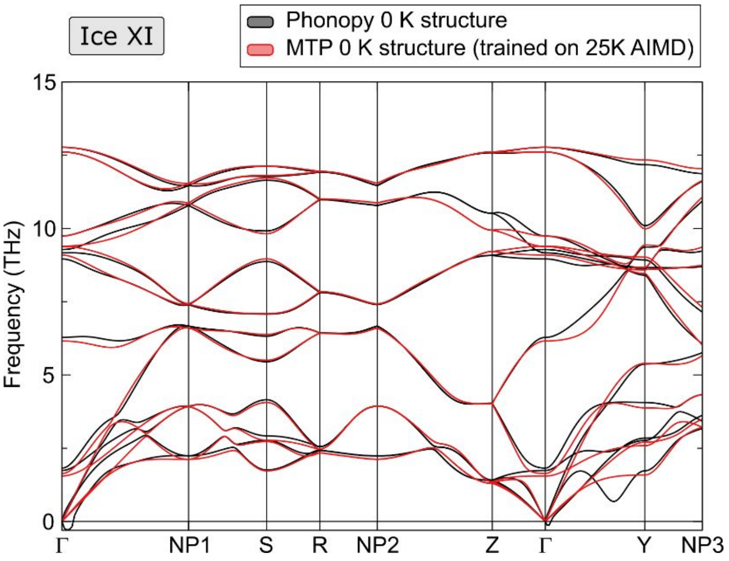

Figure S2. Comparison of calculated phonon dispersion band structure obtained from finite-difference using DFT as a force constants engine (black) and passively trained moment tensor potential as a force constant engine (red).

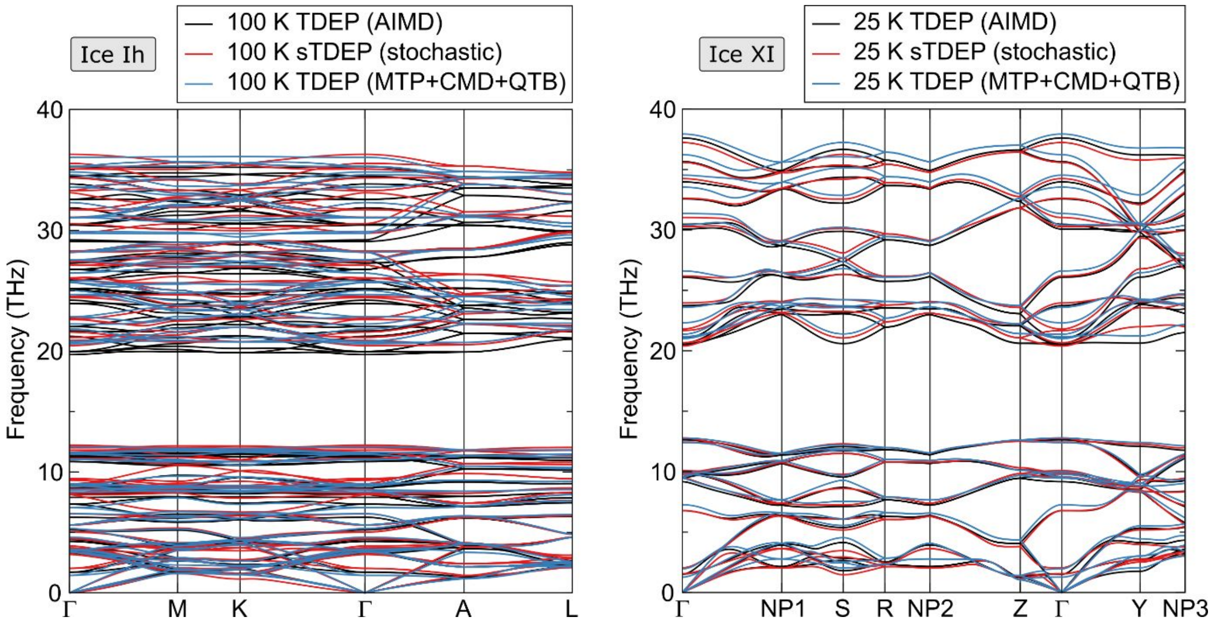

Figure S3. Calculated phonon dispersion bands using TDEP with three different ways of sampling the phase space of respective ice Ih and XI bulk phases: directly using ab-initio molecular dynamics (AIMD), sampling uncorrelated supercells within a canonical ensemble (sTDEP), and by using classical molecular dynamics coupled to a quantum thermal bath (CMD+QTB).

Surface behaviour

Table S1. Corresponding notation of low surface Miller indices between the conventional and primitive cell of ice XI.

	Conventional cell	Primitive cell
Miller index	(0 0 1)	(0 0 1)
	(0 1 0)	(1 1 0)
	(1 0 0)	(1 -1 0)
	(1 1 0)	(1 0 0)
	(1 0 1)	(1 -1 1)
	(1 1 1)	(1 0 1)
	(0 0 1)	(0 0 1)

Table S2. Calculated properties of thin surfaces of ice Ih and ice XI. Values obtained using PBE-D2.

	PBE-D2			
	Miller index	# of layers	Thickness (nm)	Surface energy (J/m^2)
Ice Ih	(1 0 0) :t0	1	0.59	0.19
		2	1.25	0.17
	(1 1 0) :t0	1	0.30	0.31
		2	0.70	0.29
	(1 1 0) :t3	1	0.38	0.34
		2	0.78	0.28
Ice XI	(0 1 0) :t0	1	0.32	0.18
		2	0.71	0.18
	(0 1 0) :t3	1	0.37	0.41
		2	0.73	0.45
	(1 0 0) :t1	1	0.16	0.43
		2	0.37	0.34
	(1 1 0) :t0	1	0.32	0.18
		2	0.70	0.19
	(1 1 0) :t3	1	0.34	0.28
		2	0.72	0.28

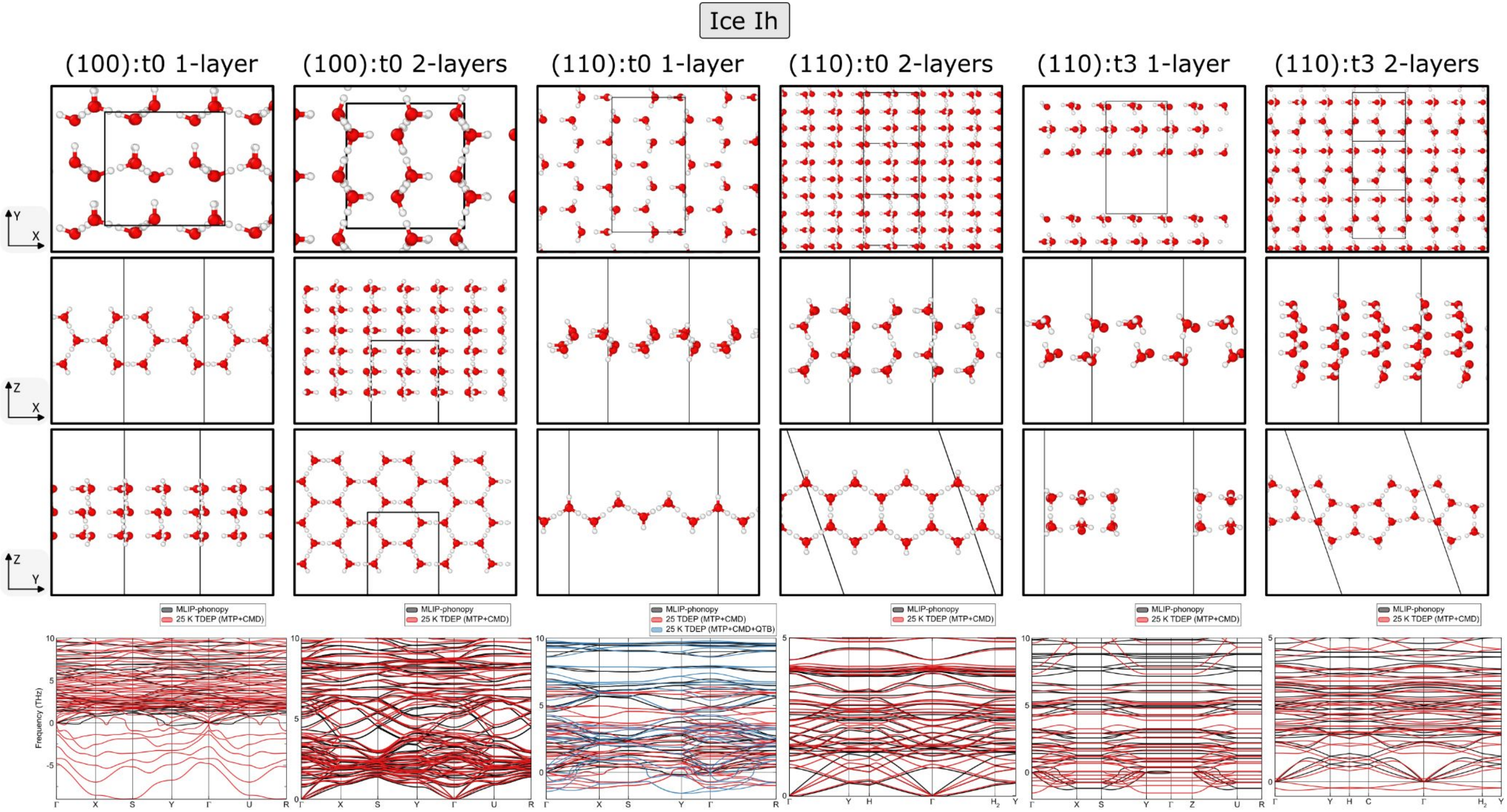

Figure S4.DFT relaxed atomic structure of thin films of ice Ih along three different crystallographic directions (top 3 rows) together with the corresponding phonon dispersion bands obtained in two different ways: directly via employing the trained MTP with the small displacement method (MLIP-phonopy) and via TDEP on top of a potential energy surfaces sampled with CMD (+QTB) simulations on top of the trained MTPs. Red spheres – oxygen, white spheres – hydrogen atoms.

Ice XI

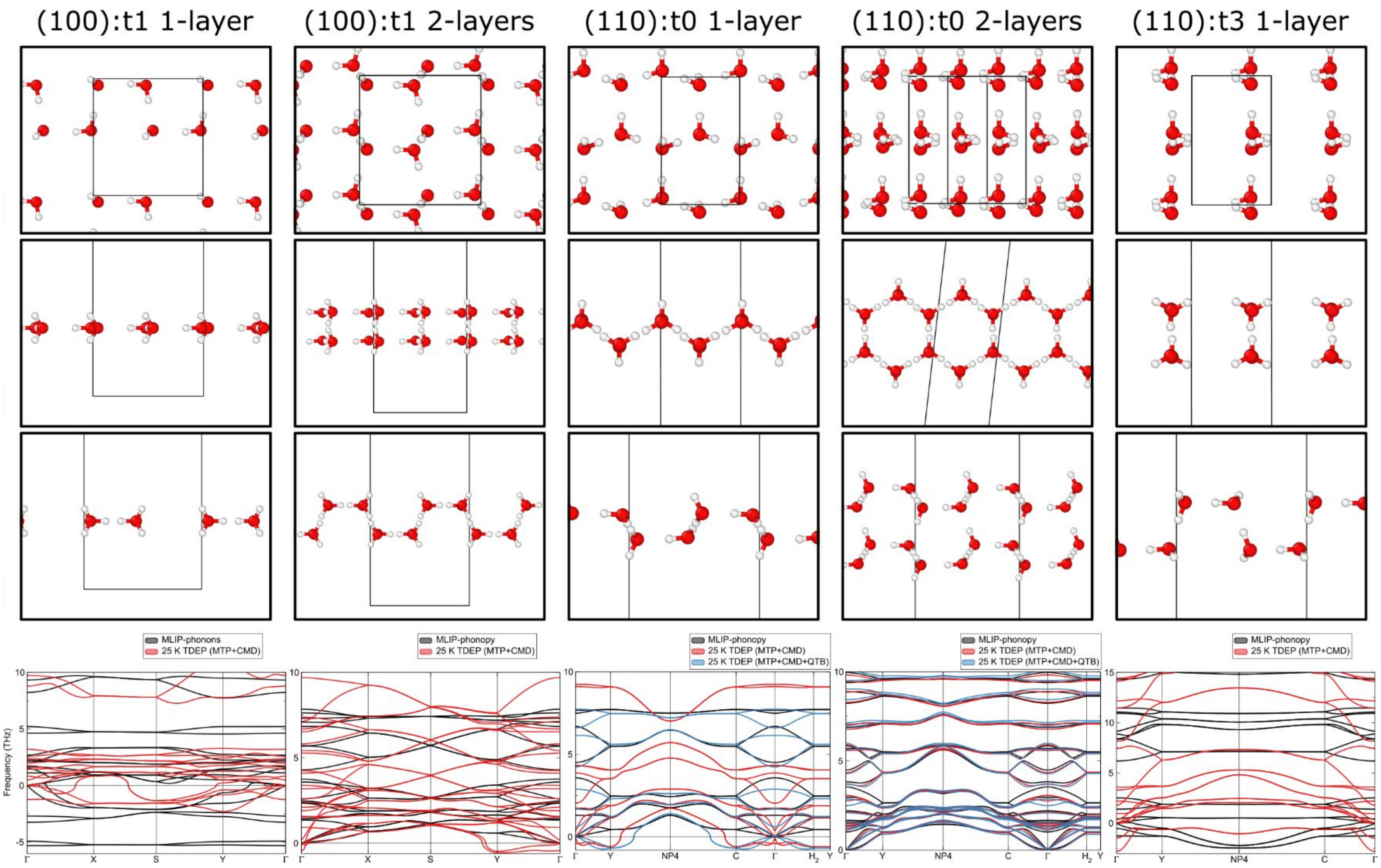

(100):t1 1-layer
(100):t1 2-layers
(110):t0 1-layer
(110):t0 2-layers
(110):t3 1-layer
MLIP-phonons
MLIP-phonopy
25 K TDEP (MTP+CMD)
25 K TDEP (MTP+CMD+QTB)
Frequency (THz)
Γ X S Y Γ
Γ Y NP4 C Γ H2 Y
Γ Y NP4 C Γ

Figure S5. DFT relaxed atomic structure of the (100) and (110) thin films of ice XI along three different crystallographic directions (top 3 rows) together with the corresponding phonon dispersion bands obtained in two different ways: directly via employing the trained MTP with the small displacement method (MLIP-phonopy) and via TDEP on top of a potential energy surfaces sampled with CMD (+QTB) simulations on top of the trained MTPs. Red spheres – oxygen, white spheres – hydrogen atoms.

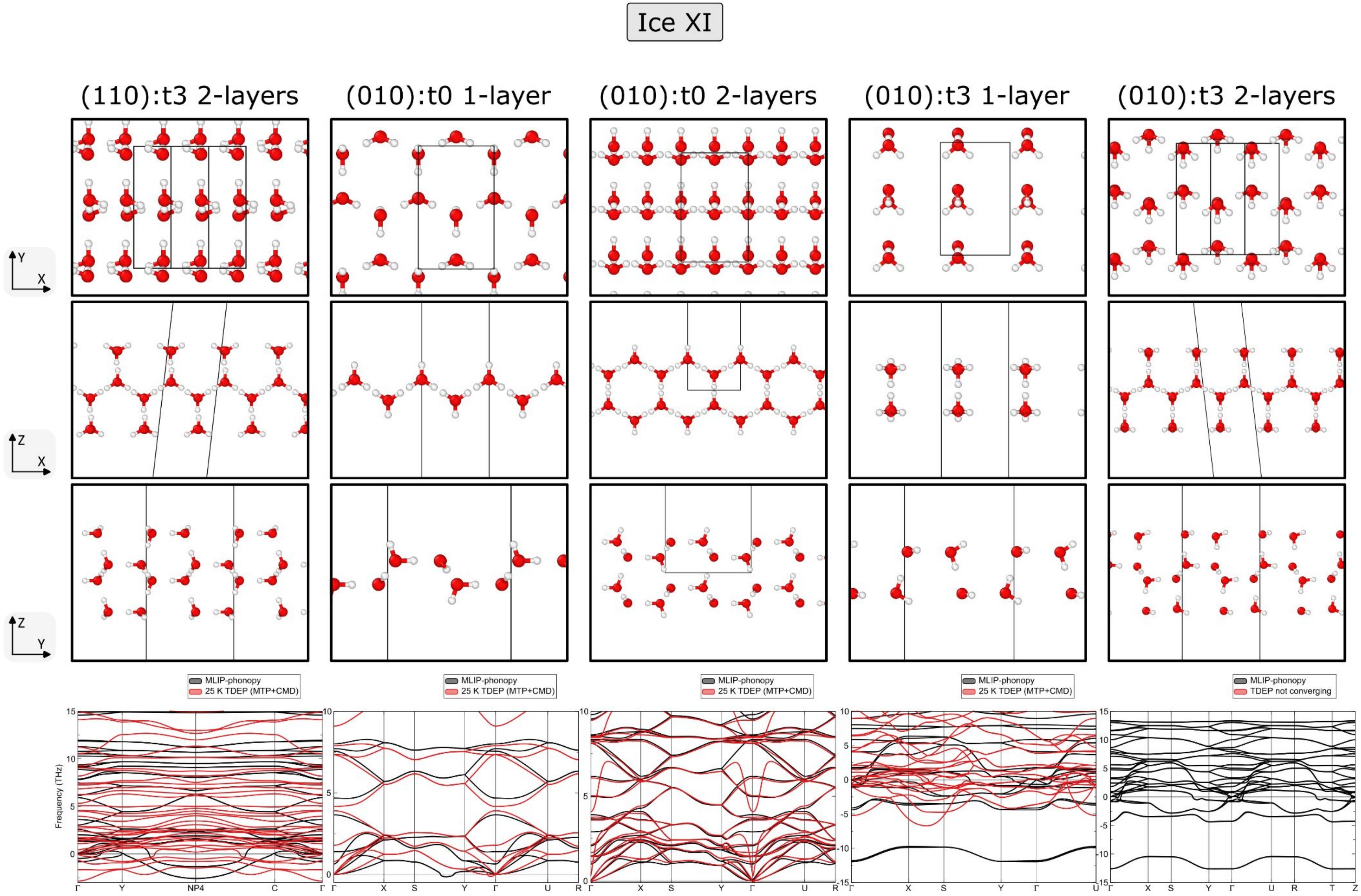

Figure S6. DFT relaxed atomic structure of the (110) and (010) thin films of ice XI along three different crystallographic directions (top 3 rows) together with the corresponding phonon dispersion bands obtained in two different ways: directly via employing the trained MTP with the small displacement method (MLIP-phonopy) and via TDEP on top of a potential energy surfaces sampled with CMD (+QTB) simulations on top of the trained MTPs. Red spheres – oxygen, white spheres – hydrogen atoms.

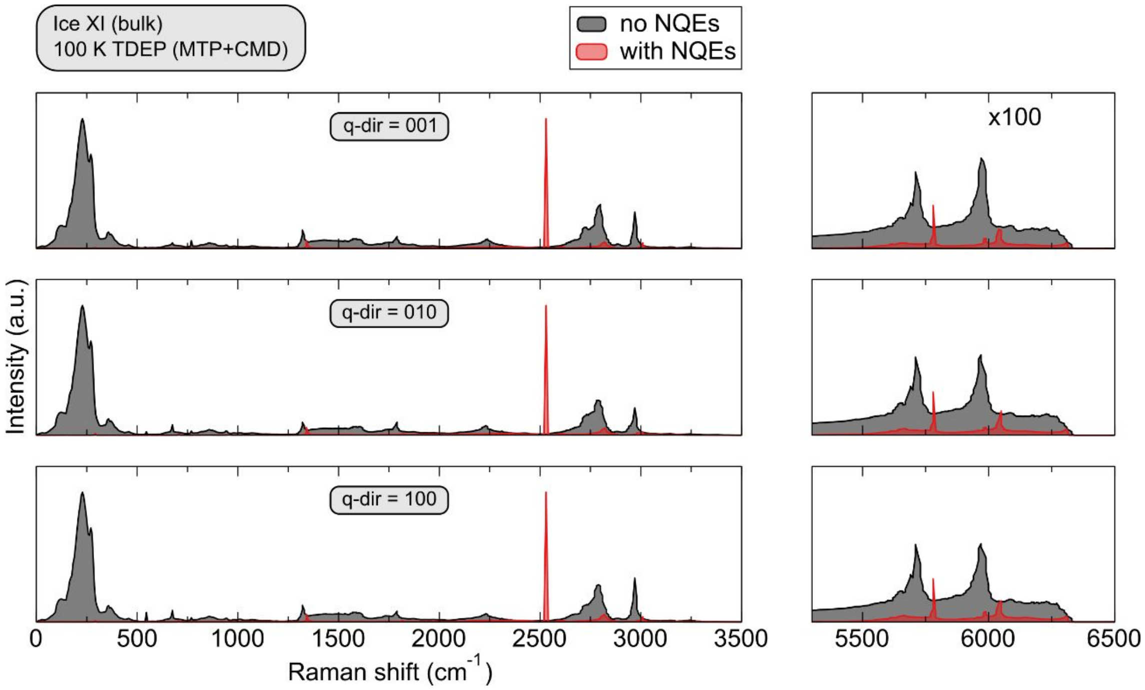

Figure S7. Computed Raman spectrum of bulk ice XI at 100 K on top of the 2nd and 3rd order force constants obtained from TDEP on top of machine learned interatomic potentials and the potential energy sampled with and without nuclear quantum effects for different incident wavevector (q-dir), which also denotes the incident laser light wrt to the crystallographic axes of the crystal. The region between 5000 cm^{-1} and 6500 cm^{-1} has been magnified for clarity.

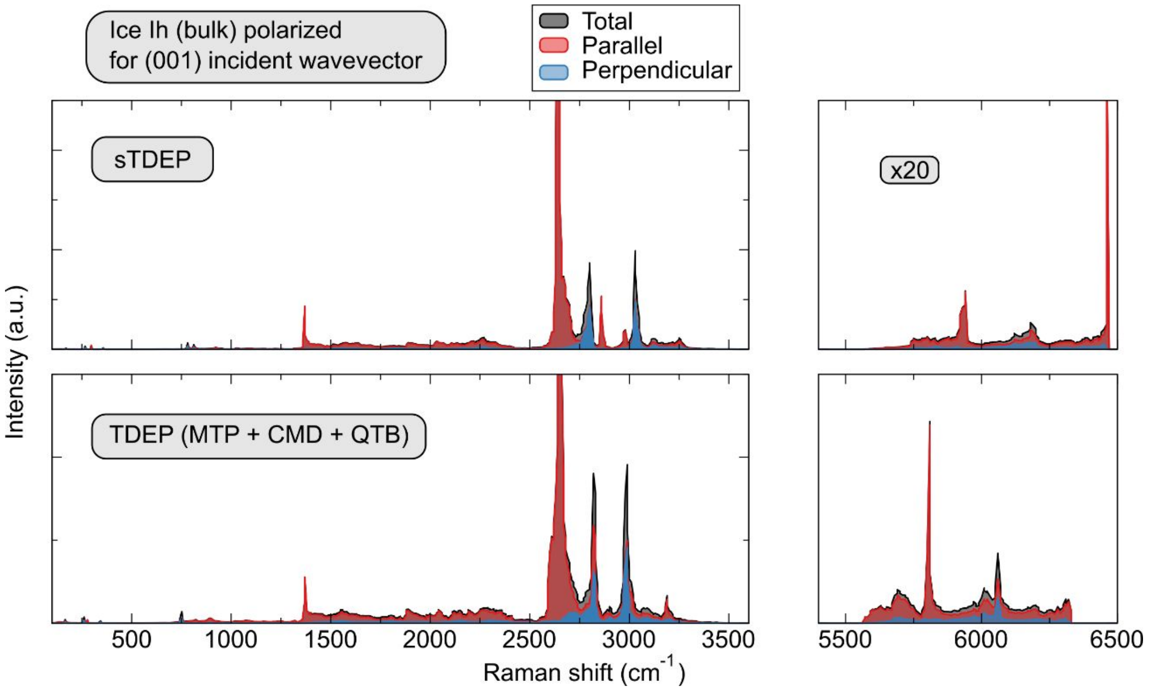

Figure S8. Computed Raman spectrum of bulk ice Ih at 100 K on top of the potential energy sampled stochastically (sTDEP) and via machine learned interatomic potentials with nuclear quantum effects (MTP+CMD+QTB). The spectra are decomposed into two polarizations: parallel and perpendicular with respect to the (001) incident wavevector.

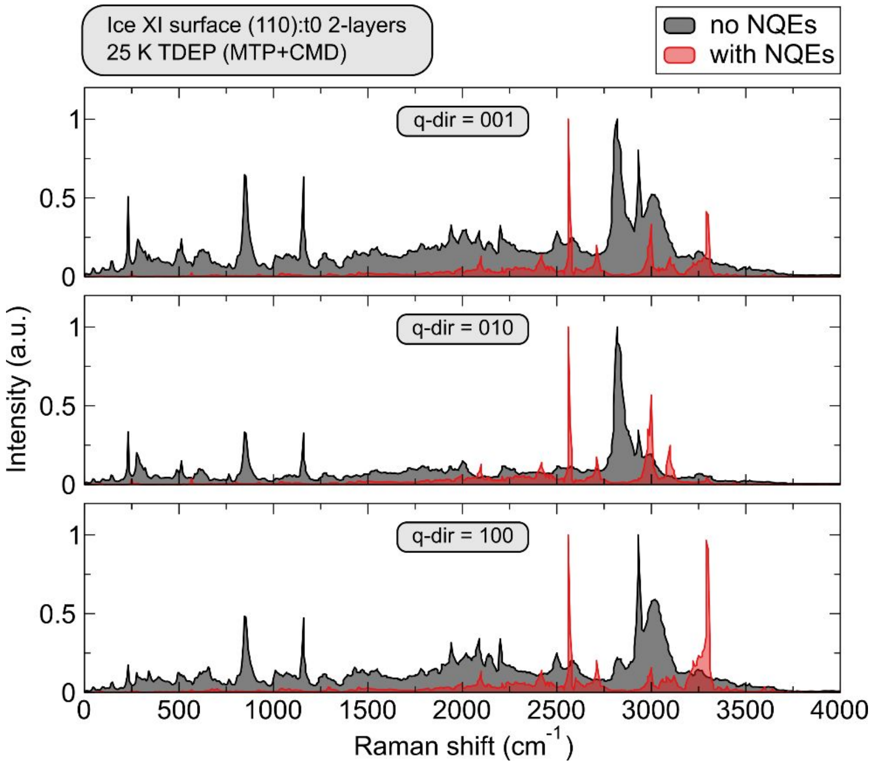

Figure S9. Computed Raman spectra of ice XI (110):t0 2-layers thin surface in contrast when NQEs are omitted and included. Values reported for three different incident wavevectors, while spectra have been normalized to unity for easier comparison.

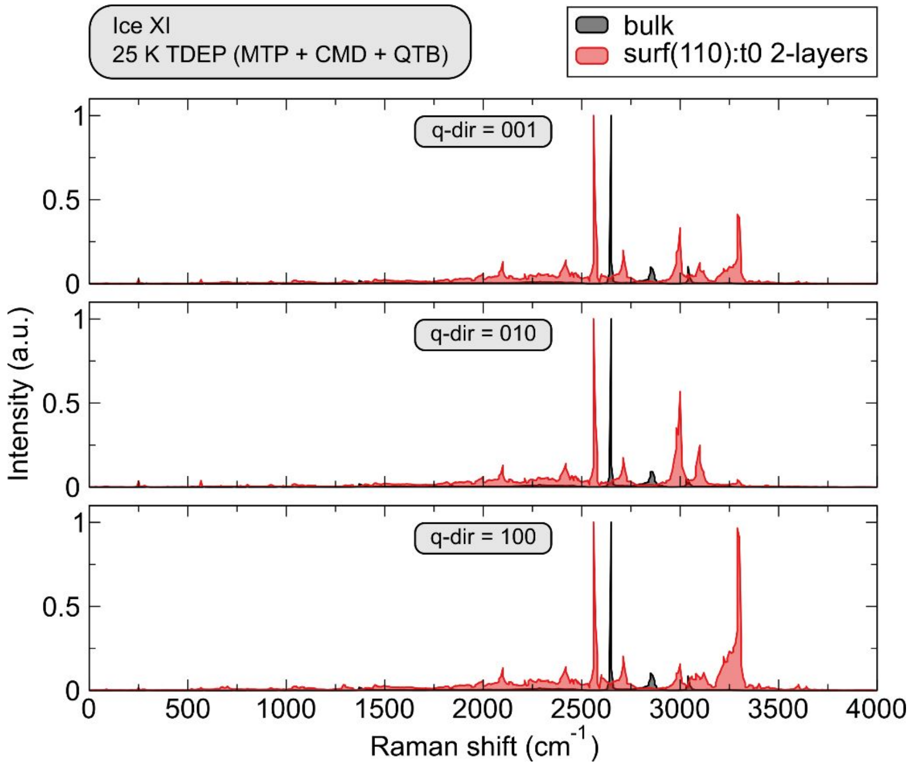

Figure S10. Computed Raman spectra of ice XI in contrast between the bulk and the (110):t0 2-layers thin surface at 25 K. Values reported for three different incident wavevectors, while spectra have been normalized to unity for easier feature identification.

Table S3. Computed elastic constants of bulk ice Ih.

									Mechanical stability criteria satisfied?			
**Phase**	**Method**	**Temp (K)**	**C_{11} (GPa)**	**C_{12} (GPa)**	**C_{13} (GPa)**	**C_{33} (GPa)**	**C_{44} (GPa)**	**C_{66} (GPa)**	**$C_{11} > 0$**	**$C_{44} > 0$**	**$C_{11}-C_{12} > 0$**	**$C_{11}+2C_{12} > 0$**
Ice Ih	LAMMPS Elastic deformation	0	25.06	14.64	10.19	21.30	3.43	5.71	Yes	Yes	Yes	Yes
	LAMMPS (MTP+CMD)	25	27.02	14.31	11.59	27.24	4.92	6.09	Yes	Yes	Yes	Yes
		50	26.90	14.30	11.59	28.82	5.32	6.79	Yes	Yes	Yes	Yes
		100	27.43	14.41	11.13	28.97	5.08	7.38	Yes	Yes	Yes	Yes
		200	27.76	13.20	10.35	26.66	6.07	6.48	Yes	Yes	Yes	Yes
		250	23.59	14.72	10.22	27.44	5.71	6.29	Yes	Yes	Yes	Yes
	LAMMPS (MTP+CMD +QTB)	25	27.13	14.01	11.33	26.86	4.92	6.06	Yes	Yes	Yes	Yes
		50	27.29	14.88	11.87	27.69	4.95	6.14	Yes	Yes	Yes	Yes
		100	27.22	14.22	12.06	28.92	5.52	6.41	Yes	Yes	Yes	Yes
		200	25.89	12.07	11.99	28.89	5.75	7.21	Yes	Yes	Yes	Yes
		250	25.28	13.06	12.62	27.36	5.46	5.64	Yes	Yes	Yes	Yes
	Exp.[3]	270	13.60	6.97	5.63	14.70	2.96	-				
	Exp. [4]	257	13.93	7.08	5.76	15.01	3.01	-				
	Exp.[5]	50	16.82	8.62	7.56	17.87	3.54	-				

Table S4. Computed elastic constants of bulk ice XI.

									Mechanical stability criteria satisfied?			
**Phase**	**Method**	**Temp (K)**	**C_{11} (GPa)**	**C_{12} (GPa)**	**C_{13} (GPa)**	**C_{33} (GPa)**	**C_{44} (GPa)**	**C_{66} (GPa)**	**$C_{11} > 0$**	**$C_{44} > 0$**	**C_{11}-$C_{12} > 0$**	**$C_{11}+2C_{12} > 0$**
Ice XI	LAMMPS Elastic deformation	0	33.94	20.00	15.19	31.82	5.69	5.21	Yes	Yes	Yes	Yes
	LAMMPS (MTP+CMD)	25	31.66	18.27	15.74	32.80	5.14	5.66	Yes	Yes	Yes	Yes
		50	31.96	18.39	16.01	32.72	5.55	6.00	Yes	Yes	Yes	Yes
		100	31.43	17.21	15.47	33.99	5.71	5.11	Yes	Yes	Yes	Yes
		200	28.15	17.96	16.11	29.29	4.88	5.72	Yes	Yes	Yes	Yes
		250	26.25	15.39	13.91	31.31	5.34	7.31	Yes	Yes	Yes	Yes
	LAMMPS (MTP+CMD +QTB)	25	31.65	18.22	15.68	32.99	5.20	5.73	Yes	Yes	Yes	Yes
		50	32.14	18.45	16.23	33.42	5.54	6.21	Yes	Yes	Yes	Yes
		100	29.85	17.13	15.22	33.15	5.85	5.62	Yes	Yes	Yes	Yes
		250	28.65	15.56	13.40	30.13	4.65	7.26	Yes	Yes	Yes	Yes

Table S5. Computed elastic constants of surfaces of ice Ih.

								Mechanical stability criteria satisfied?			
**Phase**	**Surface**	**Method**	**Temp (K)**	**C_{11} (GPa)**	**C_{12} (GPa)**	**C_{22} (GPa)**	**C_{66} (GPa)**	**$C_{11} > 0$**	**$C_{66} > 0$**	**$C_{11} > \|C_{12}\|$**	**$C_{11}C_{22} > C_{12}^2$**
Ice Ih	(100):t0 1-layer	LAMMPS Elastic deformation	0	3.52	0.77	1.97	0.64	Yes	Yes	Yes	Yes
		LAMMPS (MTP+CMD+QTB)	25	-0.91	-2.25	2.31	1.31	No	Yes	No	No
	(100):t0 2-layers	LAMMPS Elastic deformation	0	4.36	0.87	3.83	1.25	Yes	Yes	Yes	Yes
		LAMMPS (MTP+CMD+QTB)	25	5.57	1.06	4.19	1.55	Yes	Yes	Yes	Yes
	(110):t0 1-layer	LAMMPS Elastic deformation	0	16.32	-1.03	4.74	1.15	Yes	Yes	Yes	Yes
		LAMMPS (MTP+CMD+QTB)	25	3.49	0.50	2.43	0.99	Yes	Yes	Yes	Yes
	(110):t0 2-layers	LAMMPS Elastic deformation	0	5.28	1.18	7.67	2.15	Yes	Yes	Yes	Yes
		LAMMPS (MTP+CMD+QTB)	25	4.67	1.25	7.75	2.16	Yes	Yes	Yes	Yes
	(110):t3 1-layer	LAMMPS Elastic deformation	0	8.13	0.00	0.00	0.00	Yes	No	Yes	No
	(110):t3 2-layers	LAMMPS Elastic deformation	0	3.22	1.12	8.06	2.11	Yes	Yes	Yes	Yes

References

(1) Strässle, T.; Saitta, A. M.; Klotz, S.; Braden, M. Phonon Dispersion of Ice under Pressure. *Phys. Rev. Lett.* **2004**, *93* (22), 1–4. https://doi.org/10.1103/PhysRevLett.93.225901.

(2) Dorner, B. Inelastic Neutron Scattering from Ice and Other Proton-Containing Substances. *J. Glaciol.* **1978**, *21* (85), 231–240. https://doi.org/DOI: 10.3189/S0022143000033438.

(3) Gammon, P. H.; Kiefte, H.; Clouter, M. J. Elastic Constants of Ice by Brillouin Spectroscopy. *J. Glaciol.* **1980**, *25* (91), 159–168. https://doi.org/DOI: 10.3189/S0022143000010376.

(4) Gammon, P. H.; Kiefte, H.; Clouter, M. J.; Denner, W. W. Elastic Constants of Artificial and Natural Ice Samples by Brillouin Spectroscopy. *J. Glaciol.* **1983**, *29* (103), 433–460. https://doi.org/DOI: 10.3189/S0022143000030355.

(5) Neumeier, J. J. Elastic Constants, Bulk Modulus, and Compressibility of H2O Ice Ih for the Temperature Range 50 K–273 K. *J. Phys. Chem. Ref. Data* **2018**, *47* (3), 33101. https://doi.org/10.1063/1.5030640.
